# Risk and Protective Factors for Sudden Cardiac Death: An Umbrella Review of Meta-Analyses

**DOI:** 10.3389/fcvm.2022.848021

**Published:** 2022-06-16

**Authors:** Dimitrios Tsartsalis, Dafni Korela, Lars O. Karlsson, Emmanouil Foukarakis, Anneli Svensson, Aris Anastasakis, Dimitrios Venetsanos, Constantina Aggeli, Costas Tsioufis, Frieder Braunschweig, Elena Dragioti, Emmanouil Charitakis

**Affiliations:** ^1^Department of Emergency Medicine, “Hippokration” Hospital, Athens, Greece; ^2^First Department of Cardiology, “Hippokration” Hospital, University of Athens, Medical School, Athens, Greece; ^3^Department of Cardiology, Venizeleio General Hospital, Heraklion, Greece; ^4^Department of Cardiology and Department of Health, Medicine and Caring Sciences, Linköping University, Linköping, Sweden; ^5^Department of Cardiology, Onassis Cardiac Surgery Center, Athens, Greece; ^6^Department of Cardiology, Karolinska University Hospital, Stockholm, Sweden; ^7^Pain and Rehabilitation Centre and Department of Health, Medicine and Caring Sciences, Linköping University, Linköping, Sweden

**Keywords:** sudden cardiac death, risk factors, protective factors, epidemiology, meta-analysis, umbrella review

## Abstract

**Background:**

Sudden cardiac death (SCD) is a global public health issue, accounting for 10–20% of deaths in industrialized countries. Identification of modifiable risk factors may reduce SCD incidence.

**Methods:**

This umbrella review systematically evaluates published meta-analyses of observational and randomized controlled trials (RCT) for the association of modifiable risk and protective factors of SCD.

**Results:**

Fifty-five meta-analyses were included in the final analysis, of which 31 analyzed observational studies and 24 analyzed RCTs. Five associations of meta-analyses of observational studies presented convincing evidence, including three risk factors [diabetes mellitus (DM), smoking, and early repolarization pattern (ERP)] and two protective factors [implanted cardiac defibrillator (ICD) and physical activity]. Meta-analyses of RCTs identified five protective factors with a high level of evidence: ICDs, mineralocorticoid receptor antagonist (MRA), beta-blockers, and sodium-glucose cotransporter-2 (SGLT-2) inhibitors in patients with HF. On the contrary, other established, significant protective agents [i.e., amiodarone and statins along with angiotensin-converting enzyme (ACE) inhibitors in heart failure (HF)], did not show credibility. Likewise, risk factors as left ventricular ejection fraction in HF, and left ventricular hypertrophy, non-sustain ventricular tachycardia, history of syncope or aborted SCD in pediatric patients with hypertrophic cardiomyopathy, presented weak or no evidence.

**Conclusions:**

Lifestyle risk factors (physical activity, smoking), comorbidities like DM, and electrocardiographic features like ERP constitute modifiable risk factors of SCD. Alternatively, the use of MRA, beta-blockers, SGLT-2 inhibitors, and ICD in patients with HF are credible protective factors. Further investigation targeted in specific populations will be important for reducing the burden of SCD.

**Systematic Review Registration:**

https://www.crd.york.ac.uk/prospero/display_record.php?ID=CRD42020216363, PROSPERO CRD42020216363.

## Introduction

Sudden cardiac death (SCD) constitutes a significant global public health burden, with some estimates of its mortality burden as high as 20% of all deaths in industrialized countries (1–3). SCD refers to any unexpected death within 1 h of the onset of cardiac arrest symptoms.

When the death is not witnessed, the timeline expands to 24 h (4). SCD can be the first presentation of cardiovascular disease, and almost half of all SCD-victims have no previously diagnosed heart condition (1, 5).

In the past 20 years, cardiovascular mortality has decreased significantly in high-income countries (6), especially in groups with higher risk for SCD such as patients with coronary artery disease (CAD) and heart failure (HF) (7). However, recent studies from the U.S. still report a staggering incidence of cardiac arrest with over 350,000 cases out-of-hospital (3) and 290,000 in hospital (8), annually.

Identifying and targeting modifiable risk factors for SCD can improve survival for at-risk patients by preventing the onset of SCD. Yet, risk prediction for SCD is complex. The propensity for sudden death is due to1a combination of intrinsic factors, such as genetic or acquired heart diseases, and transient factors that can trigger an SCD event (7). These factors can be unmodifiable, such as age and gender or modifiable, such as ischemic heart disease (IHD), smoking, low-level physical activity, atrial fibrillation (AF), and type 2 diabetes mellitus (T2DM). Although numerous meta-analyses on risk factors of SCD have been published, there is not yet a complete and succinct summary of the research, that can be applied clinically.

Here we perform an umbrella review to summarize the existing evidence concerning risk and protective factors associated with SCD among published meta-analyses. In accordance with best research practices, we rank the evidence of existing meta-analyses in this topic according to sample size, strength of the association, and existence of diverse biases (9, 10).

## Methods

This umbrella meta-analysis was conducted according to the Preferred Reporting Items for Systematic Reviews and Meta-Analyses (PRISMA) (11) reporting guidelines and the Meta-Analysis of Observational Studies in Epidemiology (MOOSE) guidelines (12) (Appendix 1). The study protocol was registered in the prospective registry of systematic reviews, PROSPERO (CRD42020216363).

### Data Selection, Search Strategy, and Selection Criteria

We performed a systematic search in PubMed, Web of Science, Cochrane review, and Cochrane database of clinical trials through 21st May 2021, to identify systematic reviews with meta-analysis of observational or randomized controlled trials (RCT) examining associations between lifestyle factors, comorbid diseases, medications, echocardiogram (ECHO) abnormalities, electrocardiogram (ECG) abnormalities, and serum biomarkers, with the risk of SCD as a primary or secondary endpoint. Our search strategy was broad to identify all eligible studies using terms related to SCD and meta-analysis (Appendix 2). The bibliographies from eligible studies were also reviewed for identification of additional studies.

Two researchers (DK, EC) independently searched articles for eligibility. The full texts of the retrieved articles were further scrutinized for eligibility by the same researchers. Any discrepancies were resolved after consultation with a third researcher (DT).

We included only peer-reviewed systematic reviews which included meta-analyses of RCTs or observational studies with a cohort, case-control, or nested case-control study design, which measured any association between SCD and modifiable risk or protective factors, in any population. In case of the availability of multiple meta-analyses on the same topic, we proceeded with the meta-analysis with the larger number of studies, as previously described (13). All available primary and secondary reported outcomes, for each eligible meta-analysis, were considered for inclusion. Subgroup analyses are presented as reported in the original meta-analyses.

Meta-analyses were excluded if they were: (1) of other study designs than described above (i.e., cross-sectional, letter to the editor); (2) of an individual patient or participant data, pooled analyses that examined a non-systematic selection of observational studies or RCTs, and non-systematic reviews; (3) examining genetic variants as risk factors of SCD; (4) published in other languages than English; (5) provided inadequate data for quantitative synthesis; or (6) presented study-specific effects estimates as mean difference. Reasons for exclusion after full-text assessment were listed in the Appendix 3.

### Data Extraction and Quality Assessment

From each eligible article, two researchers (DK, DT), independently performed data extraction. Any disagreements were resolved by consensus. For each meta-analysis, the following variables were collected: first author, standard identifier (DOI), journal, study design, year of publication, number of component studies, total sample size, and risk and protective factors assessed. For each primary study, the following variables were collected: first author, year of publication, study design, sample size (exposure and non-exposure), and relative risk estimates [i.e., hazard ratio (HR), odds ratio (OR), risk ratio (RR)] with the corresponding 95% confidence interval (CI). The methodological quality of meta-analyses included was assessed using the AMSTAR2 (Assessment of Multiple Systematic Reviews Tool, available at https://amstar.ca/Amstar-2.php) by two independent researchers (DK, EC) (14).

### Data Synthesis and Analysis

For each association, the effect size (ES) of individual studies reported in each meta-analysis was extracted, then the pooled effect sizes and 95% confidence intervals (CIs) were re-calculated, using random-effects models (15). Inter-study heterogeneity was tested with the *I*^2^ statistic (16). Then, small-study effect bias was assessed with the Egger regression asymmetry teste and random-effects summary effect size, to determine whether smaller studies generated larger effect sizes compared with larger studies (17, 18). Finally, excess significance bias was assessed, to determine whether the observed number of studies with nominally statistically significant results was different from the expected number of studies with statistically significant results (19). The expected number of statistically significant studies per association was computed by summing the statistical power estimates for each component study. The power estimates of each component study depend on the plausible ES for the examined association, which are assumed to be the ES of the largest study (i.e., the smallest standard error) per association. For excess significance bias, a *p*-value ≤ 0.10 was considered statistically significant (19). All analyses were conducted using Stata 17.0 (StataCorp, College Station, TX) and R v.4.0.3 (The R Foundation for Statistical Computing, Auckland, NZ).

Following previous umbrella reviews (20), eligible associations from observational studies were classified into five levels, according to the strength of the evidence of potential risk or protective factors: convincing (class I), highly suggestive (class II), suggestive (class III), weak (class IV), and not significant (NS) (eTable 1, Appendix 3).

For RCTs, the credibility of evidence was classified according to the summary effect (*p*-value < 0.01, 0.01 ≤ *p*-value < 0.05, *p-*value ≥ 0.05), 95% prediction interval (excluding the null or not), and presence of large heterogeneity (*I*^2^ > 50%), small study effects (*p* < 0.10), and excess significance (*p* < 0.10) (21). An algorithm that assigns GRADE (Grading of Recommendations Assessment, Development, and Evaluation) levels of evidence (GLE) using a modified concrete set of rules was also applied (22, 23). Briefly, four areas were assessed: (1) imprecision, by the number of participants in the pooled analysis (if 100-199 participants, GLE was downgraded by 1 level; if <100 participants, downgraded by 2 levels); (2) risk of bias (RoB) trial quality, by the proportion of RCTs included in the pooled analysis with low RoB for randomization and observer blinding (if > 25% of RCTs had high RoB or RoB not reported, GLE was downgraded by 1 level); (3) inconsistency, by heterogeneity (if *I*^2^ > 75%, downgraded by 1 level); and (4) RoB review quality, by the responses to AMSTAR 2 questionnaire (if moderate quality, downgraded by 1 level; if low or critically low quality, downgraded by 2 levels). Then, reviews were classified as high, moderate, low, or very low, by GLE (eTable 2, Appendix 3).

## Results

### Literature Search

**Figure 1:**
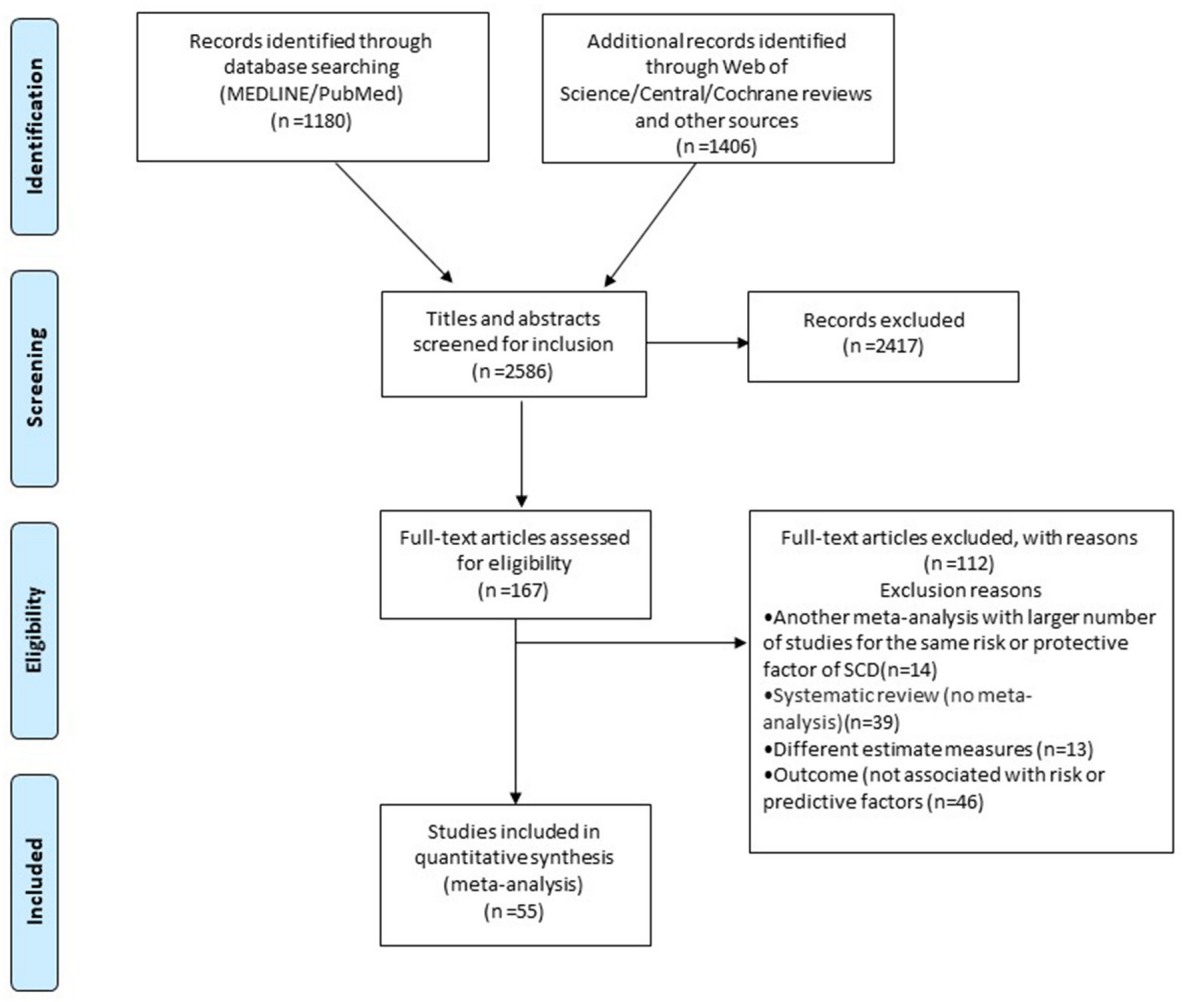
Flowchart of the study selection process (SCD, sudden cardiac death).

Initially, 2,586 publications were identified. After title and abstract screening, 167 potentially eligible articles were retrieved. Then, 112 articles were excluded after full-text assessment (Appendix 4 in the Supplementary Material). In total, 55 meta-analyses were included in the final analysis, of which 31 evaluated observational studies and reported on 83 associations, and 24 evaluated RCTs and reported on 56 associations (Figure 1; eTables 1, 2; eFigure 1 in Appendix 5).

### Meta-Analyses of Observational Studies

The quality of included meta-analyses of observational studies according to AMSTAR2 was scored as high in 10 meta-analyses, moderate in 11, and low in 10 (Appendix 5). The median number of studies included in meta-analyses was 5 (IQR = 3–8), the median number of participants was 23,839 (IQR = 5,426–78,177), and the median number of cases was 514 (IQR = 100–1,417).

In the observational studies meta-analyses, 55 of the 83 examined associations (66%) had a nominally statistically significant effect (*p* ≤ 0.05) under the random-effects models and 21 of those (38%) reached a *p*-value < 10^−6^. Thirty associations (36%) had more than 1,000 cases per association. Twenty-one associations (25%) had large heterogeneity (*I*^2^ > 50%), and only 19 associations (23%) had a 95% prediction interval that excluded the null value. In 57 associations (69%), the ES of the largest study had a nominally statistically significant effect (*p* ≤ 0.05). Finally, small-study effects were found for 12 associations (15%) and excess significance bias was found for nine associations (11%).

When the classification criteria for credibility of evidence was applied, only five (6.0%) associations presented convincing evidence (Tables 1, 3; eTable 1 in Appendix 6, Figure 1 in Appendix 7), including three risk factors (early repolarization pattern (ERP) on ECG, T2DM in general population, and smoking) and one protective (physical activity in general population). Only one intervention presented convincing evidence for its association with SCD in meta-analyses of observational studies. This intervention was the implantation of an internal cardiac defibrillator (ICD) in patients with cardiac resynchronization therapy indication (CRT), along with the CRT device. Four additional associations (4.8%) presented highly suggestive evidence for risk factors: AF, T2DM in patients with CAD, T2DM in patients with AF, and hypertension (HTN). Four associations (4.8%) presented suggestive evidence for risk factors: AF in patients with CAD, treatment with macrolides, depression, and overweight (Tables 1, 3). The remaining 42 (51%) statistically significant associations between risk or protective factors and SCD presented weak evidence (eTable 1 in Appendix 6), while 28 associations (34%) had no evidence (eTable 1 in Appendix 6).

**Table 1:** Risk and protective factors of sudden cardiac death, in meta-analyses of observational studies.

**Reference**	**Risk/Protective factor**	**Exposed/Unexposed as in included MA**	**Protective/Risk factor or intervention**	**K**	**n/N**	**Metric**	**ES (95% CI)**	***p*-value**	**PI include null value**	***I^**2**^***	**SSE**	**ESB**	**LS sign**	**CE**	**CES**	**CES2 (*n* > 1,000)**	**AMSTAR 2 Quality**
**General population**																	
Cheng (24)	Early repolarization pattern (ERP) on ECG	ERP or not	Risk	19	1,125/ 7,268	OR	4.76 (3.62, 6.26)	6.9 x 10^−29^	No	38.4%	No	NP	Yes	I	IV	I	Moderate
Aune (25)	Diabetes mellitus (DM)	DM or not	Risk	14	3,510/ 280,737	RR	2.02 (1.81, 2.25)	4.54 x 10^−37^	No	0%	No	NP	Yes	I	I	I	Moderate
Aune (26)	Smoking	Current smoker or not	Risk	4	1,061/ 203,386	RR	2.08 (1.70, 2.53)	4.85 x 10^−13^	No	17.5%	No	No	Yes	I	I	I	Moderate
Aune (27)	Physical activity	Physically active or not	Protective	8	1,193/ 136,298	RR	0.52 (0.45, 0.60)	4.77 x 10^−18^	No	0%	No	NP	Yes	I	I	I	Critically low
Rattanawong (28)	Atrial fibrillation (AF)	AF or not	Risk	28	3,258/ 75,465	RR	2.04 (1.76, 2.35)	2.83 x 10^−22^	No	43%	Yes	Yes	Yes	II	II	II	High
Pan (29)	Hypertension (HTN)	HTN or not	Risk	9	1,211/ 837,795	RR	2.1 (1.71, 2.58)	1.89 x 10^−12^	No	56.7%	No	No	Yes	II	II	II	Moderate
Cheng (30)	Macrolides	Used or not	Risk	11	58,810/ 6670,109	RR	2.42 (1.60, 3.63)	2.34 x 10^−5^	Yes	85.4%	No	No	Yes	III	NA	III	Moderate
Shi (31)	Depression	Depression or not	Risk	4	2,399/ 83,659	HR	1.98 (1.37, 2.88)	3.1 x 10^−4^	Yes	59%	Yes	Yes	Yes	III	IV	III	Critically low
Chen (32)	Body mass index (BMI)	Overweight vs. normal BMI	Risk	9	1,462/ 1188,730	RR	1.21 (1.08, 1.35)	0.001	No	7.7%	No	NP	Yes	III	III	III	Moderate
**Heart failure or LV dysfunction population**																	
Barra (33)	Implanted Cardiac defibrillator (ICD)	Eligible or not for cardiac resynchronization therapy (CRT)	Intervention	14	1,081/ 5,949	RR	0.33 (0.24, 0.47)	1.59 x 10^−10^	No	12.6%	No	NP	Yes	I	IV	I	Critically low
**Coronary artery disease population**																	
Aune (25)	DM, in patients with coronary artery disease (CAD)	DM or not	Risk	5	2,194/ 45,905	RR	1.64 (1.36, 1.97)	2.15 x 10^−7^	Yes	39.0%	Yes	No	Yes	II	II	II	Moderate
Rattanawong (28)	AF, in patients with CAD	AF or not	Risk	4	1,352/ 19,542	RR	1.56 (1.24, 1.96)	1.7 x 10^−4^	Yes	34.7%	Yes	No	Yes	III	III	III	High
**Hypertrophic cardiomyopathy population**																	
Rattanawong (28)	Atrial Fibrillation	Yes vs. No in patients with hypertrophic cardiomyopathy	Risk	4	77/ 1,662	RR	2.05 (1.22, 3.43)	0.006	Yes	25.9%	No	Yes	Yes	IV	III	IV	High quality
**Other populations**																	
Aune (25)	DM, in patients with AF, CAD, heart failure (HF), or hemodialysis	DM or not	Risk	10	2,713/ 54,735	RR	1.75 (1.51, 2.03)	1.49 x 10^−13^	No	38.6%	Yes	Yes	Yes	II	II	II	Moderate

*CE, class of evidence; CES, class of evidence sensitivity analysis; CI, confidence interval; ES, effect size; ESB, excess significance bias; HR, hazard ratio; I2, heterogeneity; K, number of studies for each factor; LS, largest study with significant effect; MA, meta-analysis; n, number of cases; N, total number of cohorts per factor; NA, not assessable; NP, not pertinent, because the number of observed studies is less than the expected; NR, not reported; OR, odds ratio; PI, prediction interval; RR, risk ratio; SSE, small study effects*.

In the prospective analysis, only three risk factors from the main analysis remained at the same class I level. These included T2DM, smoking, and physical activity. However, the five class I factors with convincing evidence in the main analysis remained convincing when associations with >1,000 cases were excluded (Table 1; eTable 1 in Appendix 6).

### Meta-Analyses of Randomized Control Trials

The quality of included meta-analyses according to AMSTAR2 was scored as high in 12 meta-analyses of RCTs, moderate in 4, and low in 8 (Appendix 5). The median number of studies included in meta-analyses of RCTs was 5.5 (IQR = 3.5–10), the median number of participants was 9,996 (IQR = 1,695–22,275), and the median number of cases was 378 (IQR = 121–700) (Table 2; eTable 2 in Appendix 6).

**Table 2:** Significant associations of interventions with the risk for sudden cardiac death, in meta-analyses of randomized controlled trials.

**Reference**	**Risk/Protective factor**	**Exposed/Unexposed as in included MA**	**K**	**n/N**	**Metric**	**ES (95% CI)**	***p*-value**	**PI include null value**	***I^**2**^***	**SSE**	**ESB**	**High RoB**	**GLE**	**AMSTAR 2 Quality**
**Heart failure or LV dysfunction population**														
Peck et al. (34)	Implantable cardioverter defibrillator (ICD) use, in patients with LV dysfunction	ICD use or not	4	261/4,269	RR	0.40 (0.31, 0.51)	4.21 x 10^13^	No	0%	No	No	<25%	High	High
Le (35)	Aldosterone antagonist use, in patients with HF	Use or not	5	456/8,301	RR	0.81 (0.67, 0.98)	0.031	Yes	7.7%	No	NP	<25%	High	High
Bapoje (36)	Mineralocorticoid receptor antagonist (MRA) use, in patients with left ventricular (LV) dysfunction	MRA use or not	6	709/11,654	OR	0.76 (0.65, 0.89)	0.001	No	0%	Yes	No	<25%	High	High
Fernandes (37)	Sodium-glucose cotransporter-2 (SGLT-2) inhibitor use, in patients with diabetes or HF	SGLT-2 use or not	8	187/45,483	OR	0.72 (0.54/0.97)	0.031	Yes	0%	No	NP	<25%	High	High
Kolodziejczak et al. (38)	ICD use, in patients with IHD and non-IHD	ICD use or conventional therapy	7	336/3,959	HR	0.41 (0.31, 0.54)	9.07 x 10^11^	No	0%	No	No	>25%	Moderate	High
Gama (39)	ICD use, in patients with HF	ICD use or not	6	1,946/2,197	RR	0.49 (0.40, 0.61)	5.46 x 10^11^	No	0%	Yes	NP	>25%	Moderate	High
Peck et al. (34)	ACEi and beta-blocker use, in patients with LV dysfunction	Use or not	10	2,824/36,172	RR	0.89 (0.81, 0.98)	0.014	Yes	31.7%	No	Yes	>25%	Moderate	High
Al-Gobari (40)	Beta-blocker use, in patients with heart failure (HF)	Beta-blocker use or not	26	1,597/24,554	OR	0.69 (0.62, 0.77)	2.79 x 10^−2^	No	0%	No	Yes	<25%	Moderate	Moderate
Chatterjee (41)	Beta-blocker use, in patients with HF	Beta-blocker use or comparator	6	787/8,960	OR	0.73 (0.63, 0.85)	3.9 x 10^5^	No	0%	No	No	<25%	Moderate	Moderate
Peck et al. (34)	MRA use, in patients with LV dysfunction using ACEi and/or beta-blockers	MRA use or not	3	691/11,032	RR	0.79 (0.68, 0.91)	0.001	Yes	0%	No	No	<25%	Moderate	High
Claro (42)	Amiodarone use, in patients with heart failure	Amiodarone use or not	11	526/4,306	RR	0.79 (0.67, 0.92)	0.004	No	0%	No	NP	>25%	Low	Critically low
**Coronary Artery disease population**														
Kolodziejczak et al. (38)	ICD use, in patients with ischemic heart disease (IHD)	ICD use or conventional therapy	4	246/2,282	HR	0.39 (0.28, 0.55)	5.95 x 10^−8^	No	0%	No	No	>25%	Moderate	High
Fernandes (43)	Trans-endocardial stem cell injection, in patients with chronic IHD	Injection or not	10	7/422	OR	0.19 (0.04, 0.86)	0.031	Yes	0%	Yes	NP	>25%	Moderate	High
Fernandes (43)	Trans-endocardial stem cell injections with other cells, in patients with chronic IHD	Injection or not	4	14/422	OR	0.24 (0.07, 0.89)	0.033	Yes	0%	No	NP	>25%	Moderate	High
Domanski (44)	Angiotensin converting enzyme inhibitor (ACEI) use, in patients with recent MI	ACEi use or not	15	900/15,103	OR	0.80 (0.70, 0.91)	0.001	No	0%	No	No	NR	Low	Critically low
Claro (42)	Amiodarone use, in post myocardial infarction (MI) patients	Amiodarone use or not	6	140/3,377	RR	0.65 (0.46, 0.91)	0.011	Yes	0%	No	NP	>25%	Low	Critically low
Zhao (45)	Omega-3 fatty acid (OFA) use, in high-incidence MI subgroup	OFA use or not	4	305/13,168	RR	0.52	0.027	Yes	33.7%	Yes	No	NR	Low	Critically low
Zhao (45)	OFA use, in low-incidence MI subgroup	OFA use or not	4	149/7,829	RR	1.39 (1.01, 1.92)	0.045	Yes	0%	No	NP	NR	Low	Critically low
Khoueiry (46)	OFA use, in patients with recent MI	OFA use or not	5	286/13,126	OR	0.69 (0.55, 0.88)	0.003	Yes	0%	No	NP	NR	Low	Critically low
**Non-ischemic cardiomyopathy population**														
Peck et al. (34)	ACEi and beta-blocker use, in patients with LV dysfunction without recent MI	Use or not	9	2,461/29,540	RR	0.91 (0.82, 1.00)	0.050	Yes	29.9%	No	No	<25%	High	High
Kolodziejczak et al. (38)	ICD use, in patients with non-ischemic heart disease (non-IHD)	ICD use or conventional therapy	3	90/1,677	HR	0.44 (0.28, 0.69)	3.41 x 10^−4^	Yes	0%	No	No	>25%	Moderate	High
Siddiqui, (47)	ICD and cardiac resynchronization therapy with ICD (CRT-D), in patients with non-IHD	CRT-D or medical management	3	90/1,677	OR	0.44 (0.28, 0.70)	0.001	Yes	0%	No	No	>25%	Moderate	High
**Hypertensive population**														
Hebert (48)	Epithelial sodium channel inhibitors combined with a thiazide diuretic	Use or not	3	100/5,761	OR	0.61 (0.39, 0.95)	0.029	Yes	0%	No	NP	NR	Low	Critically low
**High risk population for SCD**														
Claro (42)	Amiodarone use, for primary prevention	Amiodarone use or not	17	666/ 8,386	RR	0.76 (0.66, 0.88)	1.98 x 10^−4^	No	0%	No	NP	>25%	Low	Critically low
Levantesi (49)	Statin use	Statin use or not	10	688/22,275	OR	0.79 (0.67, 0.94)	0.008	Yes	9.8%	No	No	NR	Low	Critically low
**Other categories**														
Chen (50)	OFA use, in non-guidelines-adjusted therapy subgroup	OFA use or not	6	308/14,219	RR	0.67 (0.54, 0.84)	0.001	No	0%	Yes	No	<25%	Moderate	Critically low

*CE, class of evidence; CI, confidence interval; ES, effect size; ESB, excess significance bias; GLE: GRADE level of evidence; GRADE: GRADE, Grading of Recommendations Assessment, Development and Evaluation; I2, heterogeneity; K, number of studies for each factor; LS, largest study with significant effect; n, number of cases; N, total number of cohort per factor; NA, not assessable; NP, not pertinent, because the number of observed studies is less than the expected; NR, not reported; OR, odds ratio; PI, prediction interval; RoB, risk of bias; RR, risk ratio; SCD: sudden cardiac death; SSE, small study effects*.

Overall, 31 of the 56 (55%) associations reported a nominally significant summary result at *p* < 0.05 (10 had *p* < 0.001). Only 13 (23.2%) associations had a significant confidence interval, 48 (85.7%) showed no large heterogeneity (*I*^2^ < 50%), six (11%) showed small study effects, and four (7.1%) showed excess significance bias.

When the RCT credibility criteria were applied, five (8.9%) associations between protective factors and SCD presented a high GLE (Table 2). These associations included the use of MRA and ICDs in patients with left ventricular systolic dysfunction, the use of b-blockers, MRAs or angiotensin-converting enzyme (ACE) inhibitors in patients with HF, and the use of sodium-glucose cotransporter-2 (SGLT-2) inhibitors in patients with HF or DM. Twelve associations (21%) of protective factors and the risk of SCD presented a moderate GLE such as the use of beta-blockers in patients with HF, the use of ICD in patients with IHD and non-IHD, and the use of cardiac resynchronization therapy defibrillator (CRT-D) in patients with non-IHD (Tables 2, 3; eFigure 1 in Appendix 7). The remaining nine statistically significant associations (16%) between protective factors such as amiodarone and omega-3 fatty acids, with SCD presented a low GLE (Table 2), while 24 associations (43%) were not statistically significant (eTable 2 in Appendix 6).

**Table 3:** A summary of associations with high epidemiological credibility of risk and protective factors with the risk of postoperative atrial fibrillation.

**Population**	**Level of credibility**	**Factors associated with sudden cardiac death**
**General population**		
	**Meta-analyses including Observational studies**	
	*Convincing*	*Risk factors:* Early repolarization pattern, Diabetes Mellitus, and Smoking *Protective factors:* Physical activity
	*High Suggestive*	*Risk factors:* Atrial Fibrillation, and hypertension
**Heart Failure/Left ventricular**		
**dysfunction population**	**Meta-analyses including Observational studies**	
	*Convincing*	*Interventions:* Use of ICD in patients on cardiac resynchronization therapy
	**Meta-analyses including RCTs**	
	*High*	*Interventions*: Use of ICD, Sodium-glucose cotransporter-2, and mineralcorticoid receptor antagonists
	*Medium*	*Interventions*: Use of b-blockers and ACEi
**Coronary Artery disease**		
**population**	**Meta-analyses including Observational studies**	
	*Highly Suggestive*	*Risk factor:* Diabetes Mellitus
	**Meta-analyses including RCTs**	
	*Medium*	*Interventions*: Use of ICD, and trans-endocardial stem cell injection
**Non-ischemic Cardiomyopathy**		
**population**	**Meta-analyses including RCTs**	
	*High*	*Interventions*: Use of ACEi
	*Medium*	*Interventions*: Use of ICD, and CRT

*ICD, Implanted Cardiac Defibrillator; ACEi, Angiotensin converting enzyme inhibitor*.

More specifically, about the use of ICD and CRTs to prevent SCD, meta-analyses of RCTs showed that ICD prevents SCD in patients with HF, IHD, and non-IHD. The evidence of these associations was of high epidemiological credibility. However, CRT-Ds but not CRT-pacemaker (CRT-Ps) showed to protect significantly from SCD in patients with non-IHD.

## Discussion

In this study, we reviewed 55 articles concerning the risk and protective factors of SCD. Despite most of the associations being statistically significant, only a minority of them provided convincing evidence. Our meta-analyses of observational studies showed that the presence of ERP on ECG, current smoking, and T2DM were important risk factors, while physical activity was an important protective factor. In patients with HF, the use of CRT-D compared to CRT-P was the most important protective factor. Sensitivity analyses limited to prospective cohort studies did not alter marginally the main results. Our meta-analyses of RCTs showed that in patients with HF taking MRAs or SGLT-2 inhibitors, and the use of ICDs and CRT-Ds were important protective factors. Furthermore, the association of AF, HTN, and T2DM in patients with cardiovascular comorbidities with the risk of SCD was supported by highly suggestive evidence. Beta-blockers and ICDs were protective factors from SCD with moderate evidence, in certain subpopulations (Table 3).

ERP is defined as an elevation of the QRS-ST junction, J-point, and QRS notching in multiple ECG leads, and is high prevalent in middle-aged individuals (51). Although ERP in most cases can be considered benign, it is a marker of increased heterogeneity of ventricular repolarization, which might increase the risk of ventricular fibrillation (24). It is also possible that an ERP pattern can serve as a surrogate ECG marker of certain conditions known to predispose to repolarization heterogeneity, such as myocardial infarction (MI), hypokalemia, and HF. Accordingly, we found that patients with ERP are at increased risk for SCD. However, in one prospective analysis (24), ERP association with SCD was supported only by weak evidence, so future large prospective cohort studies would be of value to clarify the credibility of this association. Other electrocardiographic features, namely, the existence of premature ventricular contractions (PVCs) and microvolt T-wave alternans provided low credibility.

One of the modifiable risk factors of SCD identified in our analysis was smoking. Smoking can lead to increased blood pressure, resting heart rate, and risk of T2DM, AF, and MI, which are all risk factors of SCD (29, 52). The association between smoking and SCD can also be explained by biological mechanisms, as smoking increases the risk of ventricular arrhythmias possibly due to altered ventricular recovery time (53). Furthermore, nicotine has been shown to induce different cardiac arrhythmias in animal models, such as bradycardia, atrioventricular block, and ventricular tachyarrhythmia (54). However, other comparisons between ever, former, or never smokers and a dose-response association between smoking and SCD showed weak evidence, which may be due to the small number of patients (*n* < 1,000) in the included primary studies. Smoking as a risk factor is modifiable, and the risk of cardiovascular disease is reduced by 39% as soon as 5 years after cessation (55). Therefore, interventions targeting this risk factor may be able to have a significant impact on SCD incidence.

T2DM increased the risk for SCD by two-fold in our analysis. Several mechanisms have been postulated to explain the association between T2DM and SCD, such as myocardial disease due to atherosclerosis, inflammation-mediated associated with uremia and HTN, potassium imbalances, and arrhythmogenic effects secondary to autonomic neuropathy (56). Interventions to reduce the prevalence of T2DM, such as diet and physical activity modifications, may therefore reduce the risk of SCD indirectly. In fact, physical activity was found in our analysis to be a significant protective factor for SCD. Physical activity is important for controlling metabolic risk factors including obesity, HTN, T2DM (57), CAD (58), and HF (59), all of which are risk factors for SCD.

In patients with HF, the implantation of ICD is the most important protective factor against SCD. We found that more than two-thirds as many patients with a CRT indication are protected from SCD when they receive ICD compared to only CRT-P, with convincing evidence level. This observation is supported by other meta-analyses of RCTs, which found that ICD reduces the risk of SCD by more than half compared to standard medical treatment, in patients with reduced ejection fraction (EF). These results are consistent both in IHD patients and non-IHD patients (34, 38), with a high GLE and without significant heterogeneity.

More than ten medications have been studied for the risk of SCD, in different patient populations. Androgen deprivation therapy, macrolides, antipsychotics, and Parkinson's drugs, were evaluated in observational studies as risk factors for SCD. All were significant risk factors for SCD but, none showed high epidemiological credibility. However, in the performed sensitivity analysis, when the criterium of more than 1,000 cases per association was omitted, the association of antipsychotics with SCD was upgraded to highly convincing for risperidone and convincing for the antipsychotics' haloperidol, quetiapine, and thioridazine, a finding in line with the literature (60). The risk of SCD is high in psychiatric patients, owing to a large extent to psychotropic drugs. Different mechanisms have been introduced to explain this association (such as the increased torsadogenic effect of a psychotropic drug and the synergic effect of different proarrhythmic drugs) in the coexistence/or not of pre-existing congenital cardiopathies (such as long-QT and Brugada syndrome) (60). Predicting the safety of potential proarrhythmic medicines is a top priority (61). Thus, measures such as the use of pharmacogenetics (i.e., how genes affect the way a person responds to medications) might have relevant clinical implications, particularly for idiosyncratic adverse drug reactions, such as in the case of the use of antipsychotics and other drugs and the risk of SCD (62).

More than six medicines, including amiodarone, beta-blockers, statins, ACE-inhibitors, MRA, SGLT-2, omega-3 fatty acids, and other antihypertensive drugs were tested in meta-analyses including only RCTs. All were found to be statistically protective against SCD, but only SGLT-2 and MRA associations were supported by a high GLE. It is also important to note that these medicines are used for the treatment of heart failure (63) and arrhythmias (7) in a population already at high risk and the generalizability of these findings can be limited.

Concerning imaging-related risk factors for SCD, the presence of LGE in MRI examination was associated with SCD but was supported by weak evidence (64). The lack of strong evidence can be attributed to the small number of patients included in the original studies. When this criterium was omitted from our grading, the level of evidence was raised to convincing in patients with non-IHD. Thus, larger prospective cohort studies can be of value. Similarly, the association of the reduced LVEF with SCD was statistically significant but only supported by weak evidence in the primary analysis and highly supportive only when the criterium of *n* > 1,000 patients was omitted. This finding is surprising as low LVEF is the criterion used most commonly during the last decades to find patients eligible for ICD therapy for primary prevention of SCD. The small number of primary studies and the issue of low reproducibility of the measurement of LVEF in clinical settings can possibly explain this finding (7).

In pediatric patients with HCM traditional risk factors, such as extreme LV hypertrophy and non-sustain VT, didn't show significant associations, while others as previous history of syncope or adverse cardiac event (aborted SCD or sustain ventricular tachycardia), were significant, albeit with weak evidence. This finding could be explained by the observational study design, the small sample size, and the critically low quality of the included studies, a fact which was also annotated in the latest published guidelines (65). Hence, larger cohort studies are of great importance for optimizing risk stratification for HCM in children.

Other interesting factors associated with the risk of SCD but not included in previous meta-analyses that fulfill the inclusion criteria of our umbrella review involve risk factors such as gender in young, episodes of supraventricular tachycardias, and COVID-19 infection. Data from observational studies show that the incidence of SCD in young men is lower compared to young women, indicating that SCD due to potentially inherited cardiac diseases is less often in young women (66). Even if this factor is not modifiable, it can lead to further research about young women's protection mechanisms against SCD. SVTs have been reported to be the etiology of sudden cardiac arrest in 5% of all patients with aborted sudden death, including 7 of 13 patients without preexcitation on their baseline ECG (67). There is a subgroup of patients with SVTs with a rapid ventricular rate in which cardiac arrest may be a manifestation even in pediatric patients. Thus, electrophysiology testing must be considered, especially in the pediatric population (68). There is evidence of an increased incidence of ventricular arrhythmias and SCD in COVID-19 patients (69), while a recent meta-analysis found a higher prevalence of SCD during the COVID-19 pandemic compared to the pre-pandemic period (70). Several mechanisms have been proposed to explain the possible association of SARS-CoV-2 infection with increased SCD risk and arrhythmogenesis, including direct myocardial injury, oxygen demand-supply mismatch due to hypoxia, hypercoagulability, and adverse effects of medications for COVID-19 (71). However, reliable data assessing SARS-CoV-2 infection as a potential risk factor for SCD is still missing.

In the current guidelines (7), there is an emphasis on establishing screening and prevention programs for SCD. However, no clear recommendations for population screening have been provided due to a paucity of evidence (7). To the best of our knowledge, this is the first umbrella review providing evidence concerning the associations of modifiable risk and protective factors with the risk of SCD. Our results indicate that people who smoke tobacco and have a sedentary lifestyle, who are diagnosed with DM, AF, or HTN, as well as those who have ERP on their ECG, are at increased risk for SCD. Therefore, these factors should be considered in the design of future studies on SCD prevention. Another implication from the present study is that it identifies several protective factors such as MRA or SGLT-2 inhibitors, and the use of ICD in patients with HF. The use of them should be emphasized whenever possible in patients at high risk of SCD.

There were several modifiable risk factors with only a weak level of evidence (e.g., pre-diabetes, BMI, PVCs, etc.), in the general population and subpopulations tested. This is likely due to the limited number of available cohorts and the small number of participants available for the subpopulation analysis. Larger cohorts may be helpful for further elucidating the role of these modifiable risk factors, by providing more evidence about these associations.

Our umbrella review provides a broad picture of the non-genetic factors that have been studied for SCD. However, this study has also several limitations. First, in meta-analyses that included observational data, the associations which were supported by high epidemiological credibility can be considered strong evidence, but they cannot imply causality. On the contrary, meta-analyses which include RCTs provide data mostly in patients already at high risk, and therefore is less generalizable to the general population. Thus, our study yields risk factors with proven significant associations to SCD but does not allow conclusions as to their clinical value in primary prevention. Second, grading of meta-analyses which include observational data can provide only warnings concerning the presence of systematic biases and not proof about the nature of these biases (72, 73). Thus, only a description of the results and sources of bias has been made. Third, although a large number of risk and protective factors for SCD were included in this analysis, there may be other important factors not included, as they have not been evaluated in previously published meta-analyses, like the New York Heart Association score. In addition, potential associations of genetic factors with SCD were not assessed, as genetic causality is tested with other analytic approaches -i.e., Mendelian randomization studies- rather than pairwise meta-analysis, which was defined as the unit of analysis in the present review.

## Conclusions

In this umbrella review, we mapped the epidemiological evidence on non-genetic factors associated with SCD as identified in previously published meta-analyses. Even though SCD is a prevalent medical issue, we were only able to identify a small number of risk factors associated with SCD and even fewer with high epidemiological credibility. The association between SCD and the following risk factors were supported by convincing and highly supported evidence: lifestyle risk factors, like the lack of physical activity and smoking; comorbidities, like AF and DM; the use of medications, like MRA or SGLT-2 inhibitors; ECG features, like ERP; and the use of ICD. Further investigation with targeted interventions in these populations is the first step toward a better strategy for SCD prevention.

## Data Availability Statement

The original contributions presented in the study are included in the article/Supplementary Material, further inquiries can be directed to the corresponding author/s.

## Author Contributions

EC, DT, and ED designed the study. DK and EC performed a comprehensive screening of the literature, selected the studies included in the meta-analysis, and abstracted the data items. DT and ED performed the statistical analysis. DT and EC drafted the manuscript. EC, DT, DK, LOK, AS, DV, EF, AA, CT, CA, and FB interpreted the results and edited the manuscript critically. All the co-authors have read and accepted this version of the manuscript. All authors contributed to the article and approved the submitted version.

## Conflict of Interest

The authors declare that the research was conducted in the absence of any commercial or financial relationships that could be construed as a potential conflict of interest.

## Publisher's Note

All claims expressed in this article are solely those of the authors and do not necessarily represent those of their affiliated organizations, or those of the publisher, the editors and the reviewers. Any product that may be evaluated in this article, or claim that may be made by its manufacturer, is not guaranteed or endorsed by the publisher.
