# Supplementary material for: Risk and Protective Factors for Sudden Cardiac Death: An Umbrella Review of Meta-Analyses
Source: Front Cardiovasc Med. 2022 Jun 16;9:848021. doi: 10.3389/fcvm.2022.848021 (PMC9246322; doi:10.3389/fcvm.2022.848021)
Supplement: Supplementary file 1 [file Data_Sheet_1.docx]

**Supplementary Material**

**Risk factors and protective factors for sudden cardiac death: an umbrella review of meta-analyses**

**Appendix 1.** PRISMA 2020 and MOOSE Checklist

**Appendix 2.** Search Strategy

**Appendix 3.** Criteria for evaluation of the credibility of evidence

**Appendix 4.** List of studies excluded after full-text evaluation with reasoning

**Appendix 5.** List of studies included in the umbrella meta-analysis with baseline characteristics. Risk factors, protective factors and interventions presented according to the population exposed and the existence of statistical significance or not.

**Appendix 6.** All tested associations of interventions, and risk/protective factors with the risk of sudden cardiac death in meta-analyses of observational and randomized controlled studies

**References**

**eAppendix 1** PRISMA 2020 and MOOSE Checklist

**PRISMA 2020 Checklist**

| **Section and Topic** | **Item #** | **Checklist item** | **Location where item is reported (page)** |
| --- | --- | --- | --- |
| **TITLE** | | |  |
| Title | 1 | Identify the report as a systematic review. | 1 |
| **ABSTRACT** | | |  |
| Abstract | 2 | See the PRISMA 2020 for Abstracts checklist. | 1 |
| **INTRODUCTION** | | |  |
| Rationale | 3 | Describe the rationale for the review in the context of existing knowledge. | 1 |
| Objectives | 4 | Provide an explicit statement of the objective(s) or question(s) the review addresses. | 2 |
| **METHODS** | | |  |
| Eligibility criteria | 5 | Specify the inclusion and exclusion criteria for the review and how studies were grouped for the syntheses. | 2, 3 |
| Information sources | 6 | Specify all databases, registers, websites, organisations, reference lists and other sources searched or consulted to identify studies. Specify the date when each source was last searched or consulted. | 2, 3, Appendix 2 |
| Search strategy | 7 | Present the full search strategies for all databases, registers and websites, including any filters and limits used. | 2, 3, Appendix 2 |
| Selection process | 8 | Specify the methods used to decide whether a study met the inclusion criteria of the review, including how many reviewers screened each record and each report retrieved, whether they worked independently, and if applicable, details of automation tools used in the process. | 2, 3 |
| Data collection process | 9 | Specify the methods used to collect data from reports, including how many reviewers collected data from each report, whether they worked independently, any processes for obtaining or confirming data from study investigators, and if applicable, details of automation tools used in the process. | 2, 3 |
| Data items | 10a | List and define all outcomes for which data were sought. Specify whether all results that were compatible with each outcome domain in each study were sought (e.g. for all measures, time points, analyses), and if not, the methods used to decide which results to collect. | 2, 3 |
|  | 10b | List and define all other variables for which data were sought (e.g. participant and intervention characteristics, funding sources). Describe any assumptions made about any missing or unclear information. | 2, 3 |
| Study risk of bias assessment | 11 | Specify the methods used to assess risk of bias in the included studies, including details of the tool(s) used, how many reviewers assessed each study and whether they worked independently, and if applicable, details of automation tools used in the process. | 3 |
| Effect measures | 12 | Specify for each outcome the effect measure(s) (e.g. risk ratio, mean difference) used in the synthesis or presentation of results. | 3, 4 |
| Synthesis methods | 13a | Describe the processes used to decide which studies were eligible for each synthesis (e.g. tabulating the study intervention characteristics and comparing against the planned groups for each synthesis (item #5)). | 3, 4 |
|  | 13b | Describe any methods required to prepare the data for presentation or synthesis, such as handling of missing summary statistics, or data conversions. | 3, 4 |
|  | 13c | Describe any methods used to tabulate or visually display results of individual studies and syntheses. | 3, 4 |
|  | 13d | Describe any methods used to synthesize results and provide a rationale for the choice(s). If meta-analysis was performed, describe the model(s), method(s) to identify the presence and extent of statistical heterogeneity, and software package(s) used. | 3, 4 |
|  | 13e | Describe any methods used to explore possible causes of heterogeneity among study results (e.g. subgroup analysis, meta-regression). | 3, 4 |
|  | 13f | Describe any sensitivity analyses conducted to assess robustness of the synthesized results. | 3, 4 |
| Reporting bias assessment | 14 | Describe any methods used to assess risk of bias due to missing results in a synthesis (arising from reporting biases). | 3, 4 |
| Certainty assessment | 15 | Describe any methods used to assess certainty (or confidence) in the body of evidence for an outcome. | 3, 4 |
| **RESULTS** | | |  |
| Study selection | 16a | Describe the results of the search and selection process, from the number of records identified in the search to the number of studies included in the review, ideally using a flow diagram. | 4, Figure 1 |
|  | 16b | Cite studies that might appear to meet the inclusion criteria, but which were excluded, and explain why they were excluded. | Appendix 4 |
| Study characteristics | 17 | Cite each included study and present its characteristics. | Appendix 5 |
| Risk of bias in studies | 18 | Present assessments of risk of bias for each included study. | Appendix 5 |
| Results of individual studies | 19 | For all outcomes, present, for each study: (a) summary statistics for each group (where appropriate) and (b) an effect estimate and its precision (e.g. confidence/credible interval), ideally using structured tables or plots. | 4, 5, Table 1, 2 and Supplemental file |
| Results of syntheses | 20a | For each synthesis, briefly summarise the characteristics and risk of bias among contributing studies. | Table 1, 2 and Supplemental file |
|  | 20b | Present results of all statistical syntheses conducted. If meta-analysis was done, present for each the summary estimate and its precision (e.g. confidence/credible interval) and measures of statistical heterogeneity. If comparing groups, describe the direction of the effect. | 4, 5, Table 1, 2 and Supplemental file |
|  | 20c | Present results of all investigations of possible causes of heterogeneity among study results. | 4, 5 Table 1, 2 and Supplement file |
|  | 20d | Present results of all sensitivity analyses conducted to assess the robustness of the synthesized results. | 4, 5 Table 1, 2 and Supplement file |
| Reporting biases | 21 | Present assessments of risk of bias due to missing results (arising from reporting biases) for each synthesis assessed. | 4, 5, Table 1, 2 and Supplement file |
| Certainty of evidence | 22 | Present assessments of certainty (or confidence) in the body of evidence for each outcome assessed. | Table 1, 2 and Supplement file |
| **DISCUSSION** | | |  |
| Discussion | 23a | Provide a general interpretation of the results in the context of other evidence. | 11 |
|  | 23b | Discuss any limitations of the evidence included in the review. | 12, 13 |
|  | 23c | Discuss any limitations of the review processes used. | 12, 13 |
|  | 23d | Discuss implications of the results for practice, policy, and future research. | 12-14 |
| **OTHER INFORMATION** | | |  |
| Registration and protocol | 24a | Provide registration information for the review, including register name and registration number, or state that the review was not registered. | 2 |
|  | 24b | Indicate where the review protocol can be accessed, or state that a protocol was not prepared. | 2 |
|  | 24c | Describe and explain any amendments to information provided at registration or in the protocol. | N/A |
| Support | 25 | Describe sources of financial or non-financial support for the review, and the role of the funders or sponsors in the review. | 14 |
| Competing interests | 26 | Declare any competing interests of review authors. | 14 |
| Availability of data, code and other materials | 27 | Report which of the following are publicly available and where they can be found: template data collection forms; data extracted from included studies; data used for all analyses; analytic code; any other materials used in the review. | 14 |

MOOSE Checklist for Meta-analyses of Observational Studies

| **Reporting Criteria** | **Reported (Yes/No)** | **Reported on Page** |
| --- | --- | --- |
| **Reporting of Background** |  |  |
| Problem definition | Yes | 2 |
| Hypothesis statement | Yes | 2 |
| Description of Study Outcome(s) | Yes | 2,3 |
| Type of exposure or intervention used | Yes | 2, 3 |
| Type of study design used | Yes | 2, 3 |
| Study population | Yes | 2, 3 |
| **Reporting of Search Strategy** |  |  |
| Qualifications of searchers (eg, librarians  and investigators) | NA |  |
| Search strategy, including time period  included in the synthesis and keywords | Yes | 3, 4 and Appendix 2 |
| Effort to include all available studies,  including contact with authors | Yes | 2-4 |
| Databases and registries searched | Yes | 2 |
| Search software used, name and  version, including special features used  (eg, explosion) | NA |  |
| Use of hand searching (eg, reference  lists of obtained articles) | Yes | 2, 3 |
| List of citations located and those  excluded, including justification | Yes | Figure 1, Appendix 4, 5 |
| Method for addressing articles  published in languages other than  English | We placed restrictions on English |  |
| Method of handling abstracts and  unpublished studies | NA |  |
| Description of any contact with authors | NA |  |
| **Reporting of Methods** |  |  |
| Description of relevance or  appropriateness of studies assembled for  assessing the hypothesis to be tested | Yes | 2-4 |
| Rationale for the selection and coding of  data (eg, sound clinical principles or  convenience) | Yes | 2-4 |
| Documentation of how data were  classified and coded (eg, multiple raters,  blinding, and interrater reliability) | Yes | 2-4 |
| Assessment of confounding (eg,  comparability of cases and controls in  studies where appropriate |  | 2-4 |
| Assessment of study quality, including  blinding of quality assessors;  stratification or regression on possible  predictors of study results YES 5 |  | 2, 3 |
| Assessment of heterogeneity |  | 2-4 |
| Description of statistical methods (eg,  complete description of fixed or random  effects models, justification of whether  the chosen models account for predictors  of study results, dose-response models,  or cumulative meta-analysis) in sufficient  detail to be replicated |  | 2-4 |
| Provision of appropriate tables and  graphics |  | Figure 1, Table 1, 2 and supplement file |
| **Reporting of Results** |  |  |
| Table giving descriptive information for  each study included |  | Appendix 5 |
| Results of sensitivity testing (eg,  subgroup analysis) |  | 4-5, Table 1, 2, and supplement file |
| Indication of statistical uncertainty of  findings |  | Supplement file |
| **Reporting of Discussion** |  |  |
| Quantitative assessment of bias (eg,  publication bias) |  | NA |
| Justification for exclusion (eg, exclusion  of non–English-language citations) |  | NA |
| Assessment of quality of included studies |  | Table 1, 2, and supplement file 5-7 |
| **Reporting of Conclusions** |  |  |
| Consideration of alternative explanations  for observed results |  | 12-14 |
| Generalization of the conclusions (ie,  appropriate for the data presented and  within the domain of the literature review) |  | 12-14 |
| Guidelines for future research |  | 13-14 |
| Disclosure of funding source |  | 14 |

**Appendix 2** Search Strategy

**PubMed**

(“cardiac arrest” OR cardiac arrest [MeSH] OR “sudden cardiac death” OR sudden cardiac death[MeSH]) AND (“meta-analysis as topic”[MeSH:noexp] OR Meta-Analysis[ptyp] OR metaanaly*[tiab] OR meta-analy*[tiab])

**Web of science**

|  |  | (TS= (sudden cardiac death OR cardiac arrest) AND TS= (meta-analysis OR metaanaly* OR meta-analy*) *AND* **LANGUAGE:** (English) *AND* **DOCUMENT TYPES:** (Article) |
| --- | --- | --- |

**Cochrane review**

*Keywords:*

Sudden cardiac death

Cardiac arrest

**Cochrane central database of trials and reviews**

(sudden cardiac death OR cardiac arrest) AND (meta-analysis OR metaanaly* OR meta-analy*)

**Appendix 3** Criteria for evaluation of the credibility of evidence

eTable 1. Criteria for evaluation of the credibility of the evidence of observational studies

| **Classification** | **Criteria** |
| --- | --- |
| Convincing evidence (Class I) | 1. More than 1000 cases 2. Significant summary associations (p<1x10^-6^) per random-effects calculations 3. No evidence of small-study effects 4. No evidence of excess of significance bias 5. Prediction intervals not including the null value 6. Largest study nominally significant (p<0.05) 7. No large heterogeneity (i.e., *I^2^*< 50%) |
| Highly Suggestive evidence (Class II) | 1. More than 1000 cases 2. Significant summary associations (p<1x10^-6^) per random-effects calculation 3. Largest study nominally significant (p<0.05) |
| Suggestive Evidence (Class III) | 1. More than 1000 cases 2. Significant summary associations (p<1x10^-3^) per random-effects calculations |
| Weak evidence (Class IV) | 1. All other associations with p≤0.05 |
| Non-significant associations (NS) | 1. All associations with p >0.05 |

eTable 2. Criteria for evaluation of the credibility of the evidence of RCTs

| **Grade level of evidence** | **Number of downgrades** |
| --- | --- |
| High | 0 downgrades |
| Moderate | 1 -2 downgrades |
| Low | 3-4 downgrades |
| Very Low | 5-6 downgrades |

Note: GRADE: Grading of Recommendations Assessment,Development and Evaluation.

**Appendix 4.** List of studies excluded after full-text evaluation with reasoning

| **Author, Year** | **Reason for exclusion** |
| --- | --- |
| Neumarker, 1997^1^ | Systematic review (no meta-analysis) |
| Sim, 1997^2^ | Systematic review (no meta-analysis) |
| Piccini, 2009^3^ | Another meta-analysis with larger number of studies for the same risk or protective factor of SCD |
| Ottani, 2000^4^ | Not associated with risk or protective factor for SCD |
| Pourati, 2005^5^ | Systematic review (no meta-analysis) |
| Witte, 2005^6^ | Systematic review (no meta-analysis) |
| Lam, 2007^7^ | Systematic review (no meta-analysis) |
| Rahimi, 2012^8^ | Another meta-analysis with larger number of studies for the same risk or protective factor of SCD |
| Al-Gobari, 2017^9^ | Overlapping meta-analysis of risk or protective factors of SCD |
| Alboni, 2008^10^ | Systematic review (no meta-analysis) |
| Sousa, 2008^11^ | Not associated with risk or protective factor for SCD |
| Scott, 2009^12^ | Systematic review (no meta-analysis) |
| Cao, 2019^13^ | Meta-analysis of mean difference |
| Christiaans, 2010^14^ | Systematic review (no meta-analysis) |
| Leonardi, 2010^15^ | Systematic review (no meta-analysis) |
| Sealy, 2010^16^ | Systematic review (no meta-analysis) |
| Garritano, 2015^17^ | Systematic review (no meta-analysis) |
| Gavin, 2011^18^ | Systematic review (no meta-analysis) |
| Musa-Veloso, 2011^19^ | Another meta-analysis with larger number of studies for the same risk or protective factor of SCD |
| Lahtinen, 2012^20^ | Systematic review (no meta-analysis) |
| Rodday, 2012^21^ | Systematic review (no meta-analysis) |
| Ahn, 2017^22^ | Another meta-analysis with larger number of studies for the same risk or protective factor of SCD |
| Rossello, 2019^23^ | Another meta-analysis with larger number of studies for the same risk or protective factor of SCD |
| Bezzina, 2013^24^ | Systematic review (no meta-analysis) |
| Delise, 2018^25^ | Another meta-analysis with larger number of studies for the same risk or protective factor of SCD |
| Chen, 2013^26^ | Systematic review (no meta-analysis) |
| Chen & Hou, 2013^27^ | Another meta-analysis with larger number of studies for the same risk or protective factor of SCD |
| Liebregts, 2015^28^ | Meta-analysis of incidence rate |
| Ullal, 2016^29^ | Meta-analysis of mean difference |
| Bittencourt, 2019^30^ | Not associated with risk or protective factor for SCD |
| O'Mahony, 2019^31^ | Prevalence measured |
| Wang, 2019^32^ | ES measured |
| Zeitler, 2013^33^ | Systematic review (no meta-analysis) |
| Goldberger, 2014^34^ | Systematic review (no meta-analysis) |
| Lemaitre, 2014^35^ | Systematic review (no meta-analysis) |
| Pun, 2014^36^ | Systematic review (no meta-analysis) |
| Zaccardi, 2014^37^ | Systematic review (no meta-analysis) |
| Hernesniemi, 2015^38^ | Systematic review (no meta-analysis) |
| Kunutsor, 2016^39^ | Systematic review (no meta-analysis) |
| Ramesh, 2016^40^ | Systematic review (no meta-analysis) |
| Weng, 2016^41^ | Another meta-analysis with larger number of studies for the same risk or protective factor of SCD |
| Zhang, 2016^42^ | Systematic review (no meta-analysis) |
| Akel, 2017^43^ | Another meta-analysis with larger number of studies for the same risk or protective factor of SCD |
| Al-Khatib, 2017^44^ | Not associated with risk or protective factor for SCD |
| Narayanan, 2017^45^ | Another meta-analysis with larger number of studies for the same risk or protective factor of SCD |
| Elayi, 2017^46^ | Systematic review (no meta-analysis) |
| Di Marco, 2017^47^ | Another endpoint |
| Tereshchenko, 2017^48^ | Systematic review (no meta-analysis) |
| Xing, 2017^49^ | Another meta-analysis with larger number of studies for the same risk or protective factor of SCD |
| Alba, 2018^50^ | Meta-analysis of mean difference |
| Alba and Duero Posada, 2018^51^ | Another meta-analysis with larger number of studies for the same risk or protective factor of SCD |
| Ashar, 2018^52^ | Not associated with risk or protective factor for SCD |
| Liu, 2018^53^ | Systematic review (no meta-analysis) |
| Barra, 2018^54^ | Not associated with risk or protective factor for SCD |
| Beggs, 2018^55^ | Not associated with risk or protective factor for SCD |
| Bor, 2018^56^ | Not associated with risk or protective factor for SCD |
| Cai, 2018^57^ | Not associated with risk or protective factor for SCD |
| Simoons, 2018^58^ | Systematic review (no meta-analysis) |
| Cadrin-Tourigny, 2019^59^ | Systematic review (no meta-analysis) |
| Nalliah, 2019^60^ | Meta-analysis of Prevalence |
| Kerpen, 2019^61^ | Not associated with risk or protective factor for SCD |
| Lalande, 2019^62^ | Not associated with risk or protective factor for SCD |
| Napp, 2019^63^ | Not associated with risk or protective factor for SCD |
| Moe, 2019^64^ | Not associated with risk or protective factor for SCD |
| Osman, 2019^65^ | Not associated with risk or protective factor for SCD |
| Bazoukis, 2020^66^ | Meta-analysis of mean difference |
| Aidelsburger 2020^67^ | Another meta-analysis with larger number of studies for the same risk or protective factor of SCD |
| Cao, 2020^68^ | Meta-analysis of mean difference |
| Chahal, 2020^69^ | Systematic review (no meta-analysis) |
| Heilbrunn, 2020^70^ | Systematic review (no meta-analysis) |
| Lombardi, 2020^71^ | Systematic review (no meta-analysis) |
| Possner, 2020^72^ | Systematic review (no meta-analysis) |
| Zhu, 2020^73^ | Not associated with risk or protective factor for SCD |
| Yu, 2020^74^ | Not associated with risk or protective factor for SCD |
| Yao, 2020^75^ | Not associated with risk or protective factor for SCD |
| Turley, 2020^76^ | Not associated with risk or protective factor for SCD |
| Yan, 2020^77^ | Not associated with risk or protective factor for SCD |
| Wilson, 2020^78^ | Not associated with risk or protective factor for SCD |
| Ullah, 2021^79^ | Not associated with risk or protective factor for SCD |
| Turner, 2020^80^ | Not associated with risk or protective factor for SCD |
| Tse, 2020^81^ | Not associated with risk or protective factor for SCD |
| Tran, 2020^82^ | Not associated with risk or protective factor for SCD |
| Tian, 2020^83^ | Not associated with risk or protective factor for SCD |
| Takagi, 2020^84^ | Not associated with risk or protective factor for SCD |
| Spirito, 2021^85^ | Not associated with risk or protective factor for SCD |
| Sammani, 2020^86^ | Not associated with risk or protective factor for SCD |
| Sahu, 2020^87^ | Not associated with risk or protective factor for SCD |
| Rout, 2020^88^ | Not associated with risk or protective factor for SCD |
| Pranata, 2019^89^ | SMD measured and inclusion of cross-sectional studies |
| Rassi, 2019^90^ | Not associated with risk or protective factor for SCD |
| Rattanawong, 2019^91^ | Not associated with risk or protective factor for SCD |
| Roterberg, 2019^92^ | Another meta-analysis with larger number of studies for the same risk or protective factor of SCD |
| Vlad, 2019^93^ | Not associated with risk or protective factor for SCD |
| Yang, 2019^94^ | Not associated with risk or protective factor for SCD |
| Zang, 2019^95^ | Not associated with risk or protective factor for SCD |
| Mustafa, 2018^96^ | Not associated with risk or protective factor for SCD |
| Pickering, 2018^97^ | Not associated with risk or protective factor for SCD |
| Prins, 2018^98^ | Not associated with risk or protective factor for SCD |
| Rattanawong, 2018 | Not associated with risk or protective factor for SCD |
| Romero, 2018^99^ | Systematic review (no meta-analysis) |
| Shi, 2018^100^ | Not associated with risk or protective factor for SCD |
| Sunderland, 2017^101^ | Not associated with risk or protective factor for SCD |
| Tse, 2018^102^ | Not associated with risk or protective factor for SCD |
| Zeitler, 2018^103^ | Not associated with risk or protective factor for SCD |
| Brown, 2019^104^ | Not associated with risk or protective factor for SCD |
| Duma, 2019^105^ | Not associated with risk or protective factor for SCD |
| Lei, 2020 ^106^ | Not associated with risk or protective factor for SCD |
| Kawakami 2020^107^ | Not associated with risk or protective factor for SCD |
| Singh 2020^108^ | Letter to the editor |
| Hall 2020 ^109^ | Systematic review (no meta-analysis) |
| Couper 2020 ^110^ | Different measures used |
| Rattanawong 2020^111^ | Different measures used |
| Toloui 2021^112^ | Different measures used |

**Appendix 5.**

**eTable 1** List of studies included in the umbrella meta-analysis with baseline characteristics

| **Study** | **Type of studies included** | **Risk or protective factors of SCD** | **Exposures** | **Non-exposures (comparator)** | **No of included studies estimates** | **Sample size** | **Population (s)** | **AMSTAR 2** |
| --- | --- | --- | --- | --- | --- | --- | --- | --- |
| Claro, 2015^113^ | RCTs | Amiodarone | Amiodarone | Placebo | 17 | 9997 | Participants at high risk for SCD or participants  recovered from cardiac arrest or syncope due to ventricular arrhythmias | Critically Low Quality |
| Domanski, 1999^114^ | RCTs | ACEIs | ACEIs | Placebo | 15 | 15104 | Post-MI patients | Critically Low Quality |
| Rivero-Ayerza, 2006^115^ | RCTs | Cardiac Resynchronization Therapy | CRT | No CRT | 5 | 2371 | Patients with advanced heart failure | Critically Low Quality |
| Levantesi, 2007^116^ | RCTs | Statins | Statins | Control | 10 | 22275 | Patients at high risk of sudden cardiac death (SCD)  with evidence of atherothrombotic disease | Critically Low Quality |
| Shi, 2017^117^ | Retrospective cohort, Prospective cohort,  Case-controls | Depression | Depression | No depression | 4 | 83659 | Individuals with and without previous CVD | Critically Low Quality |
| Leon, 2008^118^ | RCTs | Fish oil | Fish oil | Placebo | 6 | 32779 | Patients with  coronary artery disease or myocardial infarction. | High Quality |
| Rafique, 2009^119^ | RCTs | Stress test | Abnormal stress test | Normal stress test | 4 | 491 | Asymptomatic patients with severe aortic stenosis | Moderate Quality |
| Skeaff, 2009^120^ | RCTs | Fish or n–3 LCPUFA | Fish or n–3 LCPUFA | Control | 6 | 52680 | Participants with or without CHD | Moderate Quality |
| Zhao, 2009^121^ | RCTs | Omega-3 fatty acids | Omega-3 fatty acids | Control | 8 | 20997 | Patients with CHD | Critically Low Quality |
| Chen, 2011^122^ | RCTs | Omega-3 fatty acids | Omega-3 fatty acids | Control | 10 | 33429 | Patients with CVD | Critically Low Quality |
| Green, 2012^123^ | Prospective cohorts | Late gadolinium enhancement on CMR | Late gadolinium enhancement | No Late gadolinium enhancement | 4 | 1063 | Patients with hypertrophic cardiomyopathy | High Quality |
| Al-Gobari, 2013^124^ | RCTs | B blockers | B blockers | Placebo | 30 | 24779 | Patients with heart failure | Moderate Quality |
| Chatterjee, 2014^125^ | RCTs | B blockers | B blockers | Placebo | 9 | 14452 | Patients with heart failure with reduced ejection fraction (HFrEF) from North America (NA) compared with other  regions of the world (ROW) | Moderate Quality |
| Khoueiry, 2013^126^ | RCTs | Omega-3 PUFAs | Omega-3 PUFAs | Control | 9 | 32919 | Patients with recent (≤6 months)  myocardial infarction.  Patients who  were scheduled for elective percutaneous coronary angioplasty  (PTCA).  Hypercholesterolemic  patients taking statins | Critically Low Quality |
| Ataklte, 2013^127^ | Retrospective cohort,  Prospective cohort,  Nested Case-controls | Premature Ventricular Complexes (PVCs) | PVCs | No PVCs | 6 | 106195 | Patients with established heart disease and General population | Critically Low Quality |
| Bapoje, 2013^128^ | RCTs | MRAs | MRAs | Control | 6 | 11875 | Patients with left  ventricular systolic dysfunction | High Quality |
| Barra, 2018^129^ | RCTs, Observational study | Cardiac Resynchronization Therapy | CRT-D | CRT-P | 14 | 18874 | CRT-D vs. CRT-P patients | Critically Low Quality |
| Hebert, 2018^130^ | RCTs | Epithelial sodium channel inhibitors combined with a thiazide diuretic | ENaC inhibitor+Thiazide diuretic | Control | 3 | 23670 | Patients with hypertension | Critically Low Quality |
| Calo, 2011^131^ | Prospective cohort | microvolt T-wave alternans | microvolt T-wave alternans | microvolt T-wave alternans | 12 | 5681 | Patients with mean age 62 years and mean ejection fraction 32% (ischemic and non-ischemic cardiomyopathi) | Moderate Quality |
| Dahabreh, 2011^132^ | Case-controls | Physical activity | Physical activity | No physical activity | 3 | 616 | Patients with MI | High Quality |
| Wu, 2016^133^ | Prospective cohort | Brugada syndrome | Brugada syndrome | No Brugada syndrome | 7 | 4494 | Patients with Brugada syndrome | High Quality |
| Rattanawong, 2018^134^ | Prospective cohort, Retrospective cohort | Atrial fibrillation | Atrial fibrillation | No atrial fibrillation | 28 | 8401 | AF population and non-AF controls | High Quality |
| Briasoulis, 2015^135^ | Prospective cohort | Myocardial fibrosis | Myocardial fibrosis | No myocardial fibrosis | 5 | 3067 | Patients with HCM | Moderate Quality |
| Norrish, 2017^136^ | Retrospective cohort | HCM and adverse events | HCM and Adverse events | HCM - no adverse events | 6 | 3394 | Patients under 18 years of age with HCM | Critically Low Quality |
| Kuruvilla, 2014^137^ | Prospective cohort | Late gadolinium enhancement/nonischemic cardiomyopathy | LGE (+) | LGE (-) | 7 | 1488 | Patients with nonischemic cardiomyopathy | High Quality |
| Peck, 2014^138^ | RCTs | Medical therapy | Active therapy | Placebo | 10 | 36172 | Patients with left ventricular systolic  dysfunction and heart failure | High Quality |
| Cheng, 2015^139^ | RCTs, Retrospective cohort, Case-controls | Macrolides | Macrolides | No macrolides | 11 | 20779963 | Patients taking macrolides and patients not on macrolide therapy | Moderate Quality |
| Cheng, 2016^140^ | Prospective cohort, Retrospective cohort, Case-controls | Early repolarization pattern | ERP (+) | ERP (-) | 8 | 334524 | General population | High Quality |
| Cheng, 2017^141^ | Prospective cohort, Retrospective cohort, Case-controls | Early repolarization pattern | ERP (+) | ERP (-) | 19 | 7268 | Patients with structural heart diseases | Moderate Quality |
| Konety, 2016^142^ | Prospective cohort | Echo predictors | \| Mitral Annular Calcification \| \| --- \| \| Aortic Sclerosis \| \| Reduced LV ejection fraction \| \| Left atrium diameter, per 1 SD \| \| Aortic root diameter, per 1 SD \| \| LV mass index, per 1 SD \| \| Mitral Peak E, per 1 SD \| \| Mitral E to A <0.70 \| \| Mitral E to A >1.5 \| | Normal Echo | 2 | 7749 | Patients with HCM, CAD, post-MI | Critically Low Quality |
| Le. 2016^143^ | RCTs | Aldosterone antagonists | Aldosterone antagonists | Control | 5 | 19333 | Patients with HF | High Quality |
| Renoux, 2016^144^ | Retrospective cohort | Dompreridone use | Dompreridone use | Control | 8 | 214962 | Patients with Parkinson’s disease | Critically Low Quality |
| Pelliccia, 2017^145^ | Prospective cohort | Hypertrophic cardiomyopathy | Obstructive hypertrophic cardiomyopathy | Nonobstructive hypertrophic cardiomyopathy | 19 | 7731 | Patients with hypertrophic cardiomyopathy | High Quality |
| Aune, 2018^146^ | Prospective cohort | Diabetes Mellitus | Diabetes Mellitus | No DM | 14 | 55098 | Patients with and without DM | Moderate Quality |
| Duan, 2015^147^ | Prospective cohort | Late gadolinium enhancement | LGE(+) | LGE (-) | 9 | 1675 | Patients with Dilated Cardiomyopathy | Moderate Quality |
| Salvo, 2016^148^ | Observational study | Antipsychotics | Antipsychotics | Placebo | 5 | 740306 | Patients with psychiatric  bipolar disorders, schizophrenia, and dementia | Critically Low Quality |
| Taverny, 2016^149^ | RCTs | Antihypertensives | Antihypertensives | Placebo | 15 | 39908 | Hypertensive individuals | High Quality |
| Kolodziejczak, 2017^150^ | RCTs | ICD therapy | ICD therapy | Conventional therapy | 7 | 8716 | Patients with ischemic and non-ischemic cardiomyopathy | High Quality |
| Siddiqui, 2018^151^ | RCTs | ICD and CRT-D | ICD and CRT-D | Medical management | 3 | 3389 | Non-ischemic cardiomyopathy patients | High Quality |
| Gama, 2020^152^ | RCTs | ICD | ICD | No ICD | 6 | 31701 | Patients with HF with reduced ejection fraction | High Quality |
| Al-Gobari, 2018^153^ | RCTs | ARBs | ARBs | Placebo | 3 | 4892 | Patients with HF with reduced ejection fraction | High Quality |
| Aune, 2018^154^ | Prospective cohort | Smoking | Smoking | No smoking | 7 | 138273 | General population | Moderate Quality |
| Aune, 2018^155^ | Prospective cohort | BMI | \| Per 5 BMI units and \| \| --- \| \| waist-to-hip ratio per 0.1 units \| | Control | 14 | 406079 | General population | High Quality |
| Ganesan, 2018^156^ | Prospective cohort, Retrospective cohort | Late gadolinium enhancement | LGE(+) | LGE (-) | 24 | 7397 | Patients with ischemic and non-ischemic  cardiomyopathy | Critically Low Quality |
| Yue, 2018^157^ | Prospective cohort | mitochondrial DNA copy number | High mtDNA copy number | Low mtDNA copy number | 2 | 29156 | Patients with and without CVD | High Quality |
| Venkatesh, 2019^158^ | Prospective cohort | Right ventricular dysfunction | Right ventricular dysfunction | No right ventricular dysfunction | 4 | 5035 | Patients after atrial switch repair | Moderate Quality |
| Fernandes, 2019^159^ | RCTs | Transendocardial stem cell injections | Transendocardial stem cell injections | Placebo | 10 | 736 | Patients with chronic ischemic heart disease | High Quality |
| Liang, 2020^160^ | RCTs, Observational Study | Androgen deprivation therapy | Androgen deprivation therapy | Placebo | 8 | 542220 | Patients with prostate cancer | High Quality |
| Chen, 2019^161^ | Prospective cohort | BMI | \| Underweight, \| \| --- \| \| Overweight, Obesity \| | Normal | 10 | 1381445 | General population | Moderate Quality |
| Pan, 2019^162^ | Prospective cohort | Hypertension | \| Hypertension, \| \| --- \| \| Systolic BP per 20mmHg,  Diastolic blood pressure (per 10 mmHg) \| | Normal | 9 | 418235 | General population | Moderate Quality |
| Yang 2020^163^ | Prospective cohort | late gadolinium enhancement (LGE) in MRI | LGE in myocarditis | No LGE in MRI | 2 | 1319 | Myocarditis | High Quality |
| Aune, 2020^164^ | Prospective cohort | Physical activity | Physical activity | No physical activity | 8 | 136298 | General population | Critically Low Quality |
| Kamp, 2020^165^ | Prospective cohort | Late gadolinium enhancement | LGE (+) | LGE (-) | 7 | 3808 | Patients with hypertrophic  cardiomyopathy | Low Quality |
| Bytyçi, 2020^166^ | Prospective cohort | Treatment technique | Surgical myectomy | Alcohol septal ablation | 7 | 4547 | Patients with obstructive Hypertrophic  Cardiomyopathy | Moderate Quality |
| Fernandes, 2021^167^ | RCT | SGLT2 inhibitors | SGLT2 inhibitors | control | 8 | 63166 | Patients with diabetes mellitus type 2 | High Quality |

Notes: BMI: Body Mass index, ACE: Angiotensin Converting enzyme; ARB: angiotensin receptor blocker BMI: Body mass index; CRT: cardiac resynchronization therapy; BP: blood pressure; ICD: Implantable cardioverter defibrillators LGE: Late gadolinium enhancement; MRA: mineralocorticoid receptor antagonist; MRI: magnetic resonance imaging; PVC: Premature Ventricular Complex; RCT: randomized controlled studies; SCD: sudden cardiac death; SD: standard deviation; SGLT-2: Sodium-glucose cotransporter-2 inhibitors (SGLT2)

eTable 2 Risk factors, protective factors and interventions that are associated with sudden cardiac death. Presented according to the population exposed and the existence of statistical significance or not

| **Population** | **Statistical Significance** | **Studied factors** |
| --- | --- | --- |
| **General population** |  |  |
|  |  |  |
|  | Statistically Significant Factors | Early repolarization pattern, Diabetes Mellitus, pre-diabetes, Smoking, (Current smoking, Former smoking, ever smoking, 10 cigarettes per day) Physical activity (physical active, episodic physical activity, per 20 metabolic equivalent (METS)-hours/day increase, per 1 metabolic equivalent (MET) increase of cardiorespiratory fitness), Atrial Fibrillation, Hypertension (yes, per 20mmHg increase), Macrolides, Depression, Body Mass Index (BMI) (overweight, per 5 BMI units, waist-to-hip ratio per 0.1 units, obesity) Premature ventricular contractions, mitochondrial DNA copy number (high) |
|  |  |  |
|  | Statistically not Significant Factors | Body mass index (BMI) (underweight, waist circumference per 10 cm increase, Diastolic Blood pressure (per 10 mmHg increase). |
| **Heart Failure or LV dysfunction population** |  |  |
|  | Statistically Significant Factors | Implanted Cardiac Defibrillator (ICD) ((yes, in patients eligible for cardiac resynchronization therapy (CRT), compared with conventional therapy), microvolt T-wave alternans, Atrial Fibrillation, Aldosterone antagonist, ACEi and beta-blocker, Sodium-glucose cotransporter-2 (SGLT-2) inhibitor, amiodarone use |
|  |  |  |
|  | Statistically not Significant Factors | cardiac resynchronization therapy (without ICD) |
| **Coronary artery disease** |  |  |
|  | Statistically Significant Factors | Diabetes Mellitus, Atrial Fibrillation, ICD (compared with conventional therapy), Trans-endocardial stem cell injection, Angiotensin converting enzyme inhibitor (ACEI) use, in patients with recent MI, Amiodarone use, in post myocardial infarction (MI) patients, Omega-3 fatty acid (OFA) (in high-incidence, low-incidence MI subgroup, recent MI) |
|  |  |  |
|  | Statistically not Significant Factors | Fish oil, Trans-endocardial stem cell injections (with skeletal myoblast, with other cells and skeletal myoblast) |
| **Hypertrophic cardiomyopathy** |  |  |
|  | Statistically Significant Factors | Atrial Fibrillation, Late gadolinium (LGE(+)), previous cardiac adverse events, syncope |
|  |  |  |
|  | Statistically not Significant Factors | obstruction, extreme hypertrophy, non-sustained ventricular arrhythmia, alcohol septal ablation |
| **Non-ischemic cardiomyopathy** |  |  |
|  | Statistically Significant Factors | Late gadolinium (LGE(+)), ACEi and beta-blocker use, in patients with LV dysfunction without recent MI, ICD, ICD and cardiac resynchronization therapy with ICD (CRT-D) |
|  |  |  |
|  | Statistically not Significant Factors |  |
| **Severe Aortic Stenosis population** |  |  |
|  | Statistically not Significant Factors | Abnormal stress test |
| **Hypertension population** |  |  |
|  | Statistically Significant Factors | Epithelial sodium channel inhibitors combined with a thiazide diuretic |
|  |  |  |
|  | Statistically not Significant Factors | Antihypertensives, Angiotensin receptor blockers (ARBs) and ACEIs, Antihypertensives (First line diuretics and other antihypertensive drugs), First line other antihypertensive drugs, First line diuretics |
| **High risk population for SCD** |  |  |
|  | Statistically Significant Factors | Amiodarone (use, primary prevention), statin |
|  |  |  |
|  | Statistically not Significant Factors | Amiodarone (vs b blockers, amiodarone for secondary prevention, vs antiarrhythmics, vs other antiarrhythmics for secondary prevention, vs other antiarrhythmics for secondary prevention in patients without ICD), omega-3 fatty acids ( in patients with history of ventricular arrhythmia and implantable cardioverter-defibrillator) |
| **Brugada Syndrome** |  |  |
|  | Statistically Significant Factors | Atrial fibrillation |
|  |  |  |
| **Myocarditis** |  |  |
|  | Statistically Significant Factors | Late gadolinium (LGE(+)) |
| **Post atrial switch repair** |  |  |
|  | Statistically not Significant Factors | Right ventricular dysfunction |
| **Other populations** |  |  |
|  | Statistically Significant Factors | Diabetes Mellitus (Patients with AF, CAD, heart failure, or hemodialysis), Atrial fibrillation (patients with internal cardiac defibrillator or pacemaker), echocardiographic factors (mitral annular calcification, left ventricular ejection fraction, left atrail diameter, left ventricular mass index in patients with cardiomyopathy or CAD), fatty acids (in non-guidelines-adjusted therapy subgroup) |
|  |  |  |
|  | Statistically not Significant Factors | echocardiographic factors (left atrial diameter per 1 SD increase, Aortic sclerosis, Mitral Peak E velocity per 1 SD increase, Mitral E to A ration>1.5, Mitral E to A ration<0.7 in patients with cardiomyopathy or CAD), omega-3 fatty acids (use, in non-guidelines-adjusted therapy subgroup), fish or n–3 PUFA (in high and low-incidence myocardial infarction subgroup, guidelines-adjusted therapy subgroup) |
| **Parkinson's disease** |  |  |
|  | Statistically Significant Factors | Domperidone in patients with vascular disease |
|  |  |  |
|  | Statistically not Significant Factors | Domperidone (use, use duration more versus use duration less than 30 days, more than 30mg, in patients without vascular disease, without concomitant use of QT prolonging drugs, mong patients with concomitant use of QT prolonging drugs) |
| **Psychiatric disease** |  |  |
|  | Statistically Significant Factors | Haloperidol, Quetiapine, Risperidone, Thioridazine |
|  |  |  |
|  | Statistically not Significant Factors | Chrorpromazine |
| **Prostate cancer** |  |  |
|  | Statistically Significant Factors | androgen deprivation therapy (with new agents, meta-analysis of RCTs) |
|  |  |  |
|  | Statistically not Significant Factors | androgen deprivation therapy (yes, with traditional anti-androgens, new hormonal agents and traditional anti-androgens) |

Notes: BMI: Body Mass index, ACE: Angiotensin Converting enzyme; ARB: angiotensin receptor blocker BMI: Body mass index; CRT: cardiac resynchronization therapy; BP: blood pressure; ICD: Implantable cardioverter defibrillators LGE: Late gadolinium enhancement; MRA: mineralocorticoid receptor antagonist; MRI: magnetic resonance imaging; PVC: Premature Ventricular Complex; RCT: randomized controlled studies; SCD: sudden cardiac death; SD: standard deviation; SGLT-2: Sodium-glucose cotransporter-2 inhibitors (SGLT2)

**eFigure 1** Forest plot of the most important risk/protective factors and interventions with the risk of sudden cardiac death from meta-analyses of observational studies


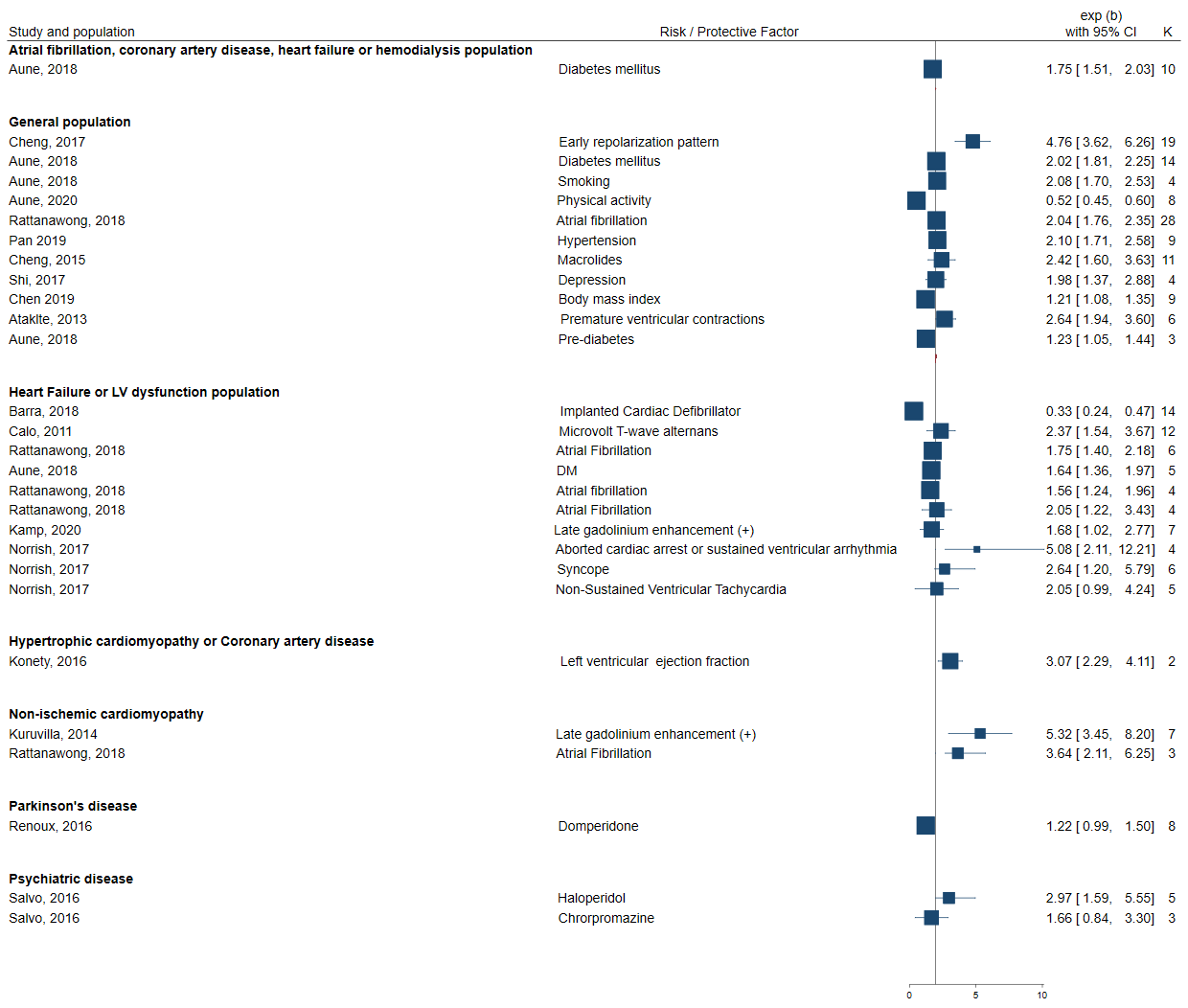


**eFigure 2** Forest plot of the most important risk/protective factors and interventions with the risk of sudden cardiac death from meta-analyses of randomized controlled studies


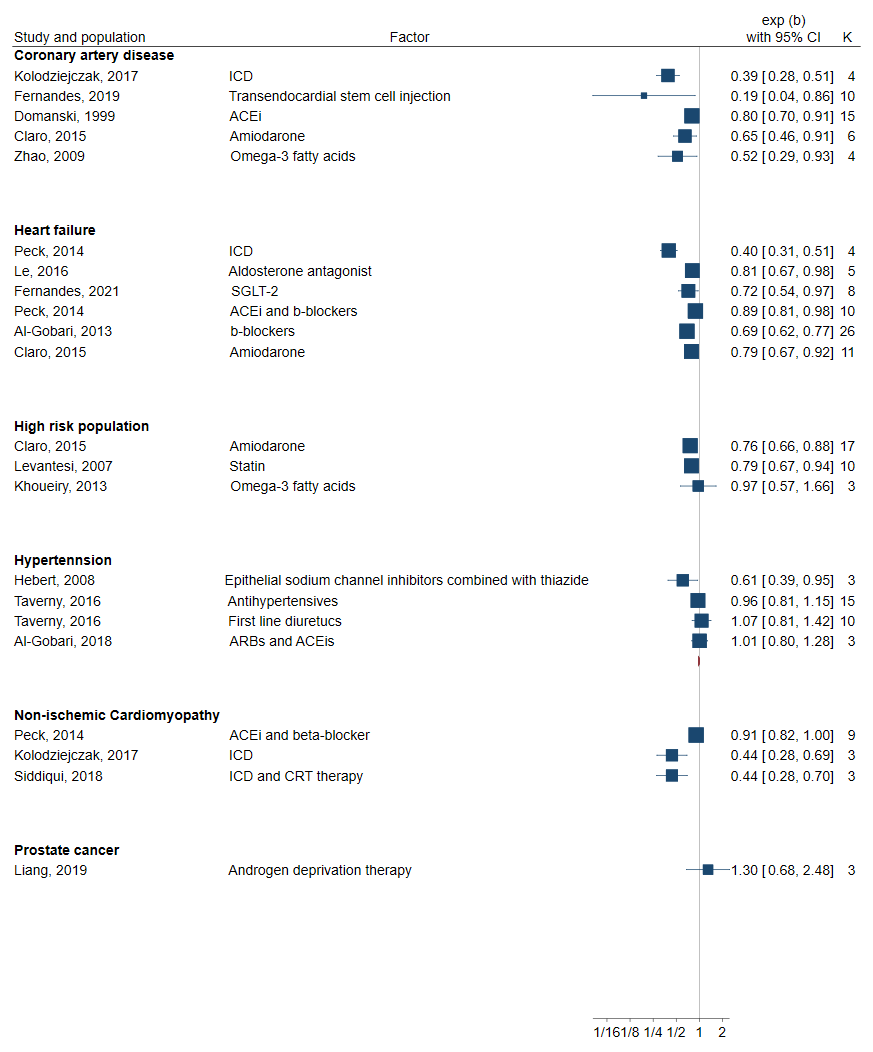


**Appendix 6** All tested associations of interventions, and risk/protective factors with the risk of sudden cardiac death in meta-analyses of observational and randomized controlled studies

eTable 1. Associations of interventions, and risk/protective factors with sudden cardiac death in meta-analyses of observational studies

| **Author, Year** | **Risk / Protective Factor** | **Exposed / Unexposed as in included MA** | **Study population** | **Protective/Risk factor** | **K** | **n / N** | **Metric** | **ES (95% CI)** | **p** | **PI include null value** | **I^2^** | **SSE** | **ESB** | **LS sign** | **CE** | **CES** | **CES2 (n>1,000)** | **AMSTAR 2 Quality** |
| --- | --- | --- | --- | --- | --- | --- | --- | --- | --- | --- | --- | --- | --- | --- | --- | --- | --- | --- |
| **General population** |  |  |  |  |  |  |  |  |  |  |  |  |  |  |  |  |  |  |
| Cheng, 2017 | Early repolarization pattern (ERP) on ECG | ERP or not | General | Risk | 19 | 1125 / 7268 | OR | 4.76 (3.62, 6.26) | 6.9 x 10^-29^ | No | 38.4% | No | NP | Yes | I | IV | I | Moderate |
| Aune, 2018 | Diabetes mellitus (DM) | DM or not | General | Risk | 14 | 3510 / 280737 | RR | 2.02 (1.81, 2.25) | 4.54 x 10^-37^ | No | 0% | No | NP | Yes | I | I | I | Moderate |
| Aune, 2018 | Smoking | Current smoker or not | General | Risk | 4 | 1061 / 203386 | RR | 2.08 (1.70, 2.53) | 4.85 x 10^-13^ | No | 17.5% | No | No | Yes | I | I | I | Moderate |
| Aune, 2020 | Physical activity | Physically active or not | General | Protective | 8 | 1193 / 136298 | RR | 0.52 (0.45, 0.60) | 4.77 x 10^-18^ | No | 0% | No | NP | Yes | I | I | I | Critically low |
| Rattanawong, 2018 | Atrial fibrillation (AF) | AF or not | General | Risk | 28 | 3258 / 75465 | RR | 2.04 (1.76, 2.35) | 2.83 x 10^-22^ | No | 43% | Yes | Yes | Yes | II | II | II | High |
| Pan 2019 | Hypertension (HTN) | HTN or not | General | Risk | 9 | 1211 / 837795 | RR | 2.1 (1.71, 2.58) | 1.89 x 10^-12^ | No | 56.7% | No | No | Yes | II | II | II | Moderate |
| Cheng, 2015 | Macrolides | Used or not | General | Risk | 11 | 58810 / 6670109 | RR | 2.42 (1.60, 3.63) | 2.34 x 10^-5^ | Yes | 85.4% | No | No | Yes | III | NA | III | Moderate |
| Shi, 2017 | Depression | Depression or not | General | Risk | 4 | 2399 / 83659 | HR | 1.98 (1.37, 2.88) | 3.1 x 10^-4^ | Yes | 59% | Yes | Yes | Yes | III | IV | III | Critically low |
| Chen 2019 | Body mass index (BMI) | Overweight vs normal BMI | General | Risk | 9 | 1462 / 1188730 | RR | 1.21 (1.08, 1.35) | 0.001 | No | 7.7% | No | NP | Yes | III | III | III | Moderate |
| Ataklte, 2013 | Premature ventricular contractions (PVCs) | Frequent PVCs versus infrequent PVCs | General | Risk | 6 | 394/42351 | RR | 2.64 (1.94, 3.60) | 8.7x10^-10^ | No | 0% | No | NP | Yes | IV | IV | I | Critically low quality |
| Dahabreh, 2011 | Episodic physical activity | Episodic Physical activity versus no physical activity | General | Risk | 3 | 648/ 106645 | RR | 4.98 (1.47, 16.91) | 0.010 | Yes | 94.7% | No | No | Yes | IV | NA | IV | High quality |
| Aune, 2018 | pre-diabetes | Yes versus No | General | Risk | 3 | 1000/ 18360 | RR | 1.23 (1.05, 1.44) | 0.012 | Yes | 6.2% | No | No | Yes | IV | IV | IV | Moderate quality |
| Aune, 2018 | Smoking | current smoking vs never smoking | General | Risk | 7 | 770/ 40180 | RR | 3.06 (2.46, 3.82) | 2.90x10^-23^ | No | 40.5% | No | No | Yes | IV | IV | I | Moderate quality |
| Aune, 2018 | Smoking | former smoking vs never smoking | General | Risk | 7 | 717/ 40180 | RR | 1.38 (1.20 1.60) | 9.88x10^-6^ | No | 0.0% | No | No | Yes | IV | IV | III | Moderate quality |
| Aune, 2018 | Smoking | ever smoking vs never smoking | General | Risk | 7 | 993/ 75597 | RR | 2.01 (1.70 2.38) | 5.53x10^-6^ | No | 54.1% | No | No | Yes | IV | IV | III | Moderate quality |
| Aune, 2018 | Smoking | Per 10 cigarettes/day increase | General | Risk | 2 | 469/ 108764 | RR | 1.58 | 2.46x10^-12^ | NA | 0.0% | NA | No | Yes | IV | IV | II | Moderate quality |
| Aune, 2018 | Body mass index (BMI) | per 5 BMI units increase | General | Risk | 14 | 2394/ 371882 | RR | 1.16 (1.05, 1.28) | 0.003 | Yes | 67.7% | Yes | No | Yes | IV | IV | IV | High quality |
| Aune, 2018 | Body mass index (BMI) | Waist-to-hip ratio per 0.1 units increase | General | Risk | 3 | 543/ 170101 | RR | 1.82 (1.61, 2.06) | 6.61x10^-21^ | Yes | 0.0% | No | No | Yes | IV | IV | II | High quality |
| Yue, 2018 | mitochondrial DNA copy number | high versus low | General | Risk | 2 | 3919/ 7837 | OR | 1.83 (1.22, 2.74) | 0.004 | NA | 61.5% | NA | No | Yes | IV | IV | IV | High quality |
| Chen 2019 | Body mass index (BMI) | obesity vs normal | General | Risk | 10 | 985/ 591129 | RR | 1.53 (1.31, 1.77) | 3.81x10^-8^ | No | 29.7% | No | NP | Yes | IV | IV | I | Moderate quality |
| Pan 2019 | Hypertension - | Per 20mmHg increase | General | Risk | 9 | NA/NA | RR | 1.28 (1.18, 1.38) | 2.53x10^-10^ | No | 44.7% | No | NP | Yes | IV | IV | I | Moderate quality |
| Aune, 2020 | Physical activity | per 20 metabolic equivalent (METS)-hours/day increase | General | Protective | 3 | 581/ 57813 | RR | 0.67 (0.53, 0.86) | 0.001 | Yes | 39.0% | No | No | Yes | IV | IV | III | Critically Low Quality |
| Aune, 2020 | Physical activity | Per 1 metabolic equivalent (MET) increase of cardiorespiratory fitness | General | Protective | 2 | 362/ 57813 | RR | 0.54 (0.36, 0.79) | 0.002 | NA | 0.0% | NP | No | Yes | IV | IV | IV | Critically Low Quality |
| Aune, 2018 | Body mass index (BMI) | Waist circumference per 10 cm increase | General | Risk | 2 | 151/ 12015 | RR | 1.03 (0.92, 1.14) | 0.539 | NA | 0.0% | NA | NP | No | NS | NA | NA | High quality |
| Chen 2019 | Body mass index (BMI)-Underweight patients | underweight vs normal BMI | General | Risk | 3 | 100/ 150697 | RR | 1.20 (0.95, 1.51) | 0.132 | Yes | 0.0% | No | NP | No | NS | NA | NA | Moderate quality |
| Pan 2019 | Diastolic blood pressure- | per 10 mmHg increase | General | Risk | 3 | NA/NA | RR | 1.09 (0.83, 1.43) | 0.537 | Yes | 83.1% | No | NP | Yes | NS | NA | NA | Moderate quality |
| **Heart Failure or LV dysfunction population** |  |  |  |  |  |  |  |  |  |  |  |  |  |  |  |  |  |  |
| Barra, 2018 | Implanted Cardiac Defibrillator (ICD) | Eligible or not for cardiac resynchronization therapy (CRT) | HF or/and left ventricular dysfunction | Intervention | 14 | 1081 / 5949 | RR | 0.33 (0.24, 0.47) | 1.59 x 10^-10^ | No | 12.6% | No | NP | Yes | I | IV | I | Critically low |
| Calo, 2011 | microvolt T-wave alternans | Abnormal versus Normal | HF/LV dysfunction, >62 years | Risk | 12 | 489/ 4600 | RR | 2.37 (1.54, 3.67) | 9.7x10^-5^ | Yes | 45.8% | Yes | NP | No | IV | IV | III | Moderate quality |
| Rattanawong, 2018 | Atrial Fibrillation | Yes versus No in patients with congestive heart failure | HF/LV dysfunction | Risk | 6 | 399/ 7748 | RR | 1.75 (1.40, 2.18) | 8.25x10^-7^ | No | 0.0% | No | NP | Yes | IV | II | I | High quality |
| **Coronary artery disease** |  |  |  |  |  |  |  |  |  |  |  |  |  |  |  |  |  |  |
| Aune, 2018 | DM, in patients with coronary artery disease (CAD) | DM or not | CAD | Risk | 5 | 2194 / 45905 | RR | 1.64 (1.36, 1.97) | 2.15 x 10^-7^ | Yes | 39.0% | Yes | No | Yes | II | II | II | Moderate |
| Rattanawong, 2018 | AF, in patients with CAD | AF or not | CAD | Risk | 4 | 1352 / 19542 | RR | 1.56 (1.24, 1.96) | 1.7 x 10^-4^ | Yes | 34.7% | Yes | No | Yes | III | III | III | High |
| **Hypertrophic cardiomyopathy** |  |  |  |  |  |  |  |  |  |  |  |  |  |  |  |  |  |  |
| Rattanawong, 2018 | Atrial Fibrillation | Yes versus No in patients with hypertrophic cardiomyopathy | Hypertrophic cardiomyopathy | Risk | 4 | 77/1662 | RR | 2.05 (1.22, 3.43) | 0.006 | Yes | 25.9% | No | Yes | Yes | IV | III | IV | High quality |
| Kamp, 2020 | Late gadolinium (LGE(+)) enhancement in patients with HC | Yes versus No in Hypertrophic cardiomyopathy | Hypertrophic cardiomyopathy | Risk | 7 | 80/ 3708 | OR | 1.68 (1.02, 2.77) | 0.040 | Yes | 0.0% | No | NP | No | IV | IV | IV | Low quality |
| Norrish, 2017 | Previous cardiac adverse events (aborted cardiac arrest or sustained ventricular arrhythmia) | Yes versus No in patients with childhood hypertrophic cardiomyopathy | Childhood hypertrophic cardiomyopathy | Risk | 4 | 68/ 451 | OR | 5.08 (2.11, 12.21) | 2,8x10^-4^ | Yes | 0.0% | No | NP | Yes | IV | NA | II | Critically Low Quality |
| Norrish, 2017 | syncope | Yes, versus No in patients with childhood hypertrophic cardiomyopathy | Childhood hypertrophic cardiomyopathy | Risk | 6 | 92/561 | OR | 2.64 (1.20, 5.79) | 0.015 | Yes | 40.7% | No | No | No | IV | NA | IV | Critically Low Quality |
| Pelliccia, 2017 | obstructive hypertrophic cardiomyopathy | obstructive hypertrophic cardiomyopathy versus non-obstructive hypertrophic cardiomyopathy | Hypertrophic cardiomyopathy | Risk | 19 | 420/ 7335 | RR | 0.81 (0.60, 1.09) | 0.166 | Yes | 50.1% | No | Yes | No | NS | NA | NA | High quality |
| Norrish, 2017 | Non-Sustained Ventricular Tachycardia | Yes, versus No in patients with childhood hypertrophic cardiomyopathy | Childhood hypertrophic cardiomyopathy | Risk | 5 | 119/ 605 | OR | 2.05 (0.99, 4.24) | 0.054 | Yes | 9.6% | No | NP | No | NS | NA | NA | Critically Low Quality |
| Norrish, 2017 | Extreme left ventricular hypertrophy (LVH) | Yes, versus No in patients with childhood hypertrophic cardiomyopathy | Childhood hypertrophic cardiomyopathy | Risk | 3 | 100/ 428 | OR | 1.70 (0.85, 3.40) | 0.136 | Yes | 30.7% | No | NP | Yes | NS | NA | NA | Critically Low Quality |
| Bytyçi 2020 | alcohol septal ablation | alcohol septal ablation vs surgical myectomy | Obstructive Hypertrophic Cardiomyopathy | Risk | 7 | 17/ 1336 | RR | 1.66 (0.65, 4.22) | 0.290 | Yes | 0.0% | Yes | NP | No | NS | NA | NA | Moderate quality |
| Green, 2012 | late gadolinium enhancement (LGE) on CMR | Yes, versus no in hypertrophic cardiomyopathy | hypertrophic cardiomyopathy | risk | 4 | 22/ 1063 | OR | 2.40 (0.87, 6.59) | 0.090 | Yes | 0.2% | No | NP | No | NS | NA | NA | High quality |
| **Non-ischemic cardiomyopathy** | | | |  |  |  |  |  |  |  |  |  |  |  |  |  |  |  |
| Kuruvilla, 2014 | Late gadolinium enhancement (LGE(+)) in CMR | Yes, versus No in patients with non-ischemic cardiomyopathy | Non-ischemic cardiomyopathy | Risk | 7 | 133/ 1194 | OR | 5.32 (3.45, 8.20) | 3.68x10^-14^ | No | 0.0% | No | NP | Yes | IV | NA | I | High quality |
| Duan, 2015 | Late gadolinium (LGE (+)) enhancement in dilated cardiomyopathy | Yes, versus No in dilated cardiomyopathy | Non ischemic cardiomyopathi | Risk | 9 | 40/1285 | OR | 3.29 (1.73, 6.25) | 2.77x10^-4^ | No | 0.0% | No | NP | Yes | IV | IV | III | Moderate quality |
| **Brugada Syndrome** |  |  |  |  |  |  |  |  |  |  |  |  |  |  |  |  |  |  |
| Rattanawong, 2018 | Atrial Fibrillation | Yes, versus No in patients with brugada syndrome | Brugada syndrome | Risk | 3 | 446/ 6285 | RR | 3.64 (2.11, 6.25) | 3.08x10^-6^ | Yes | 0.0% | No | No | Yes | IV | NS | II | High quality |
| **Myocarditis** | |  |  |  |  |  |  |  |  |  |  |  |  |  |  |  |  |  |
| Yang, 2020 | late gadolinium enhancement LGE (+) in CMR | Yes, versus No in myocarditis | Myocarditis | Risk | 2 | 75/ 482 | OR | 7.32 (0.27, 202.15) | 0.240 | NA | 62.7% | NA | NP | Yes | NS | NA | NA | High quality |
| **Post atrial switch repair** |  |  |  |  |  |  |  |  |  |  |  |  |  |  |  |  |  |  |
| Venkatesh, 2019 | Right ventricular dysfunction | Yes, versus No | Post atrial switch repair | Risk | 4 | 12/ 349 | OR | 2.76 (0.81, 9.41) | 0.106 | Yes | 0.0% | No | NP | No | NS | NA | NA | Moderate quality |
| **Other populations** |  |  |  |  |  |  |  |  |  |  |  |  |  |  |  |  |  |  |
| Aune, 2018 | DM, in patients with AF, CAD, heart failure (HF), or hemodialysis | DM or not | Specific populations (AF or CAD or HF or hemodialysis) | Risk | 10 | 2713 / 54735 | RR | 1.75 (1.51, 2.03) | 1.49 x 10^-13^ | No | 38.6% | Yes | Yes | Yes | II | II | II | Moderate |
| Rattanawong, 2018 | Atrial Fibrillation | Yes versus No in patients with internal cardiac defibrillators (ICD) and pacemaker | Patients with internal cardiac defibrillators (ICD) and pacemaker | Risk | 4 | 209/ 4860 | RR | 1.83 (1.23, 2.71) | 0.003 | Yes | 16.9% | No | Yes | No | IV | IV | IV | High quality |
| Konety, 2016 | Echocardiographic predictors: Mitral Annular Calcification | Yes versus No | Hypertrophic cardiomyopathy or CAD | Risk | 2 | 72/6125 | HR | 1.85 (1.36, 2.52) | 8.7x10^-5^ | NA | 0.0% | NA | No | Yes | IV | IV | III | Critically Low Quality |
| Konety, 2016 | Echocardiographic predictors: Left ventricular (LV) ejection fraction | Reduced versus normal | Hypertrophic cardiomyopathy or CAD | Risk | 2 | 40/7464 | HR | 3.07 (2.29, 4.11) | 4.19x10^-14^ | NA | 0.0% | NA | NP | Yes | IV | IV | II | Critically Low Quality |
| Konety, 2016 | Echocardiographic predictors: Left atrium diameter | per 1 SD increase | Hypertrophic cardiomyopathy or CAD | Risk | 2 | 8/7123 | HR | 1.15 (1.02, 1.30) | 0.025 | NA | 0.0% | NA | NP | No | IV | IV | IV | Critically Low Quality |
| Konety, 2016 | Echocardiographic predictors: LV mass index, | per 1 SD increase | Hypertrophic cardiomyopathy or CAD | Risk | 2 | 50/5426 | HR | 1.30 (1.15, 1.48) | 5.6x10^-5^ | NA | 0.0% | NA | NP | Yes | IV | IV | III | Critically Low Quality |
| Konety, 2016 | Echocardiographic predictors/Aortic Sclerosis | Yes versus No | Hypertrophic cardiomyopathy, CAD, post-MI | Risk | 2 | 62/6025 | HR | 1.22 (0.79, 1.89) | 0.367 | NA | 22.6% | NA | NP | No | NS | NA | NA | Critically Low Quality |
| Konety, 2016 | Echocardiographic predictors: Left atrium diameter | per 1 SD increase | Hypertrophic cardiomyopathy, CAD, post-MI | Risk | 2 | 6/ 7352 | HR | 1.11 (0.96, 1.28) | 0.149 | NA | 13.4% | NA | NP | No | NS | NA | NA | Critically Low Quality |
| Konety, 2016 | Echocardiographic predictors /Mitral Peak E velocity | per 1 SD increase | Hypertrophic cardiomyopathy, CAD, post-MI | Risk | 2 | 2/ 7388 | HR | 1.13 (0.92, 1.38) | 0.253 | NA | 56.7% | NA | No | No | NS | NA | NA | Critically Low Quality |
| Konety, 2016 | Echo predictors/Mitral E to A ratio<0.70 | Yes versus No | Hypertrophic cardiomyopathy, CAD, post-MI | Risk | 2 | 80/ 1217 | HR | 0.69 (0.10, 4.79) | 0.711 | NA | 91.3% | NA | No | Yes | NS | NA | NA | Critically Low Quality |
| Konety, 2016 | Echo predictors/Mitral E to A ration>1.5 | Yes versus No | Hypertrophic cardiomyopathy, CAD, post-MI | Risk | 2 | 29/453 | HR | 1.51 (0.81, 2.83) | 0.198 | NA | 34.4% | NA | No | Yes | NS | NA | NA | Critically Low Quality |
| **Parkinson's disease** | |  |  |  |  |  |  |  |  |  |  |  |  |  |  |  |  |  |
| Renoux, 2016 | Domperidoneuse | Use versus no use in Parkinson's disease among patient with vascular disease | Parkinson's disease among patient with vascular disease | Risk | 8 | 1768/31212 | RR | 1.38 (1.07, 1.78) | 0.015 | Yes | 0.0% | No | NP | No | IV | NA | IV | Critically Low Quality |
| Renoux, 2016 | Domperidone use | Use vs no use in Parkinson's disease | Parkinson’s | Risk | 8 | 2753/ 78177 | RR | 1.22 (0.99, 1.50) | 0.059 | Yes | 0.0% | Yes | NP | No | NS | NA | NA | Critically Low Quality |
| Renoux, 2016 | Domperidone | use duration more versus use duration less than 30 days in Parkinson's disease | Parkinson’s disease | Risk | 5 | 2323/ 68279 | RR | 1.61 (0.73, 3.56) | 0.243 | Yes | 49.2% | Yes | NP | No | NS | NA | NA | Critically Low Quality |
| Renoux, 2016 | Domperidone | Dose more than 30mg or not in Parkinson's disease | Parkinson’s disease | Risk | 7 | 2697/ 77084 | RR | 1.17 (0.86, 1.59) | 0.304 | Yes | 0.0% | Yes | NP | No | NS | NA | NA | Critically Low Quality |
| Renoux, 2016 | Domperidone | use versus no use in Parkinson's disease among patient without vascular disease | Parkinson’s disease | Risk | 7 | 973/47029 | RR | 1.34 (0.80, 2.25) | 0.266 | Yes | 28.2% | No | NP | No | NS | NA | NA | Critically Low Quality |
| Renoux, 2016 | Domperidone | use versus no use in Parkinson's disease among patients without concomitant use of QT prolonging drugs | Parkinson’s disease | Risk | 8 | 1371/ 52128 | RR | 1.40 (0.85, 2.31) | 0.184 | Yes | 21.1% | No | NP | No | NS | NA | NA | Critically Low Quality |
| Renoux, 2016 | Domperidone | use vs no use in Parkinson's disease among patients with concomitant use of QT prolonging drugs | Parkinson’s | Risk | 8 | 1370/ 26361 | RR | 1.22 (0.93, 1.60) | 0.146 | Yes | 0.0% | No | NP | No | NS | NA | NA | Critically Low Quality |
| **Psychiatric disease** | |  |  |  |  |  |  |  |  |  |  |  |  |  |  |  |  |  |
| Salvo, 2016 | Antipsychotics/ Haloperidol | Use versus no use | Psychiatric disease | Risk | 5 | 132/ | OR | 2.97 (1.59, 5.55) | 0.001 | Yes | 76.2% | No | No | Yes | IV | NA | III | Critically Low Quality |
| Salvo, 2016 | Antipsychotics/ Quetiapine | Use versus no use | Psychiatric disease | Risk | 3 | 57/ 23839 | OR | 1.72 (1.33, 2.23) | 3.80x10^-4^ | Yes | 0.0% | No | No | Yes | IV | NA | III | Critically Low Quality |
| Salvo, 2016 | Antipsychotics/ Risperidone | Use versus no use | Psychiatric disease | Risk | 3 | 115/ 29043 | OR | 3.03 (2.39, 3.85) | 9.68x10^-20^ | Yes | 0.0% | No | No | Yes | IV | NA | II | Critically Low Quality |
| Salvo, 2016 | Antipsychotics/ Thioridazine | Use versus no use | Psychiatric disease | Risk | 3 | 91/ 15892 | OR | 4.58 (2.09 10.05) | 1.48x10^-4^ | Yes | 50.3% | Yes | No | Yes | IV | NA | III | Critically Low Quality |
| Salvo, 2016 | Antipsychotics/ Chrorpromazine | Use versus no use | Psychiatric disease | Risk | 3 | 40/ 3207 | OR | 1.66 (0.84, 3.30) | 0.148 | Yes | 0.0% | No | NP | No | NS | NA | NA | Critically Low Quality |
|  |  |  |  |  |  |  |  |  |  |  |  |  |  |  |  |  |  |  |
| **Prostate cancer** | |  |  |  |  |  |  |  |  |  |  |  |  |  |  |  |  |  |
| Liang, 2019 | androgen deprivation therapy | Yes versus No with new hormonal agents | Prostate cancer | Risk | 6 | 857/ 8074 | RR | 1.28 (1.01, 1.62) | 0.044 | Yes | 68.2% | No | NP | Yes | IV | NA | IV | High quality |
| Liang, 2019 | androgen deprivation therapy | Yes versus No | Prostate cancer | Risk | 8 | 23504/ 358117 | RR | 1.13 (0.93, 1.38) | 0.225 | Yes | 78.1% | No | NP | Yes | NS | NA | NA | High quality |
| Liang, 2019 | androgen deprivation therapy with traditional anti-androgens | Yes versus No | Prostate cancer | Risk | 5 | 30492/ 279000 | RR | 1.02 (0.60, 1.74) | 0.929 | Yes | 99.0% | No | NP | Yes | NS | NA | NA | High quality |
| Liang, 2019 | androgen deprivation therapy with new hormonal agents and traditional anti-androgens | Yes versus No | Prostate cancer | Risk | 11 | 31349/ | RR | 1.15 | 0.412 | Yes | 97.6% | No | NP | Yes | NS | NA | NA | High quality |

Notes: CE, class of evidence; CI, confidence interval; ES, effect size; ESB, excess significance bias; I2, heterogeneity; K, number of studies for each factor; LS, largest study with significant effect; n, number of cases; N, total number of cohort per factor; NA, not assessable; NR, not reported; NP, not pertinent because the number of observed studies is less than the expected; OR, odds ratio; RR, risk ratio; PI, prediction interval; SSE, small study effects; sign., significant; eOR>1 indicates risk factor.

eTable 2. Associations of interventions with the risk of sudden cardiac death in meta-analyses of randomized control trials

| **Author, Year** | **Risk / Protective Factor** | **Exposed / Unexposed as in included MA** | **K** | **n / N** | **Metric** | **ES (95% CI)** | **p** | **PI include null value** | **I^2^** | **SSE** | **ESB** | **GLE** | **AMSTAR 2 Quality** |
| --- | --- | --- | --- | --- | --- | --- | --- | --- | --- | --- | --- | --- | --- |
| **Heart Failure or LV dysfunction population** |  |  |  |  |  |  |  |  |  |  |  |  |  |
| Peck, 2014 | Implantable cardioverter defibrillator (ICD) use, in patients with LV dysfunction | ICD use or not | 4 | 261 / 4,269 | RR | 0.40 (0.31, 0.51) | 4.21 x 10^-13^ | No | 0% | No | No | High | High |
| Le, 2016 | Aldosterone antagonist use, in patients with HF | Use or not | 5 | 456 / 8,301 | RR | 0.81 (0.67, 0.98) | 0.031 | Yes | 7.7% | No | NP | High | High |
| Bapoje, 2013 | Mineralocorticoid receptor antagonist (MRA) use, in patients with left ventricular (LV) dysfunction | MRA use or not | 6 | 709 / 11,654 | OR | 0.76 (0.65, 0.89) | 0.001 | No | 0% | Yes | No | High | High |
| Fernandes, 2021 | Sodium-glucose cotransporter-2 (SGLT-2) inhibitor use, in patients with diabetes or HF | SGLT-2 use or not | 8 | 187 / 45,483 | OR | 0.72 (0.54/0.97) | 0.031 | Yes | 0% | No | NP | High | High |
| Kolodziejczak, 2017 | ICD use, in patients with IHD and non-IHD | ICD use or conventional therapy | 7 | 336 / 3,959 | HR | 0.41 (0.31, 0.54) | 9.07 x 10^-11^ | No | 0% | No | No | Moderate | High |
| Gama, 2020 | ICD use, in patients with HF | ICD use or not | 6 | 1,946 / 2,197 | RR | 0.49 (0.40, 0.61) | 5.46 x 10^-11^ | No | 0% | Yes | NP | Moderate | High |
| Peck, 2014 | ACEi and beta-blocker use, in patients with LV dysfunction | Use or not | 10 | 2,824 / 36,172 | RR | 0.89 (0.81, 0.98) | 0.014 | Yes | 31.7% | No | Yes | Moderate | High |
| Al-Gobari, 2013 | Beta-blocker use, in patients with heart failure (HF) | Beta-blocker use or not | 26 | 1,597 / 24,554 | OR | 0.69 (0.62, 0.77) | 2.79 x 10^-12^ | No | 0% | No | Yes | Moderate | Moderate |
| Chatterjee, 2014 | Beta-blocker use, in patients with HF | Beta-blocker use or comparator | 6 | 787 / 8,960 | OR | 0.73 (0.63, 0.85) | 3.9 x 10^-5^ | No | 0% | No | No | Moderate | Moderate |
| Peck, 2014 | MRA use, in patients with LV dysfunction using ACEi and/or beta-blockers | MRA use or not | 3 | 691  /  11,032 | RR | 0.79 (0.68, 0.91) | 0.001 | Yes | 0% | No | No | Moderate | High |
| Claro, 2015 | Amiodarone use, in patients with heart failure | Amiodarone use or not | 11 | 526 / 4,306 | RR | 0.79 (0.67, 0.92) | 0.004 | No | 0% | No | NP | Low | Critically low |
| Rivero-Ayerza, 2006 | cardiac resynchronization therapy | Yes versus no in advanced HF and a depressed left ventricular systolic performance | 5 | 147/2371 | OR | 1.04 (0.73, 1.48) | 0.843 | Yes | 0.0% | No | NP | NS | Critically Low Quality |
| **Coronary Artery disease population** |  |  |  |  |  |  |  |  |  |  |  |  |  |
| Kolodziejczak, 2017 | ICD use, in patients with ischemic heart disease (IHD) | ICD use or conventional therapy | 4 | 246 / 2,282 | HR | 0.39 (0.28, 0.55) | 5.95 x 10^-8^ | No | 0% | No | No | Moderate | High |
| Fernandes, 2019 | Trans-endocardial stem cell injection, in patients with chronic IHD | Injection or not | 10 | 7 /  422 | OR | 0.19 (0.04, 0.86) | 0.031 | Yes | 0% | Yes | NP | Moderate | High |
| Fernandes, 2019 | Trans-endocardial stem cell injections with other cells, in patients with chronic IHD | Injection or not | 4 | 14 / 422 | OR | 0.24 (0.07, 0.89) | 0.033 | Yes | 0% | No | NP | Moderate | High |
| Domanski, 1999 | Angiotensin converting enzyme inhibitor (ACEI) use, in patients with recent MI | ACEi use or not | 15 | 900 / 15,103 | OR | 0.80 (0.70, 0.91) | 0.001 | No | 0% | No | No | Low | Critically low |
| Claro, 2015 | Amiodarone use, in post myocardial infarction (MI) patients | Amiodarone use or not | 6 | 140 / 3,377 | RR | 0.65 (0.46, 0.91) | 0.011 | Yes | 0% | No | NP | Low | Critically low |
| Zhao, 2009 | Omega-3 fatty acid (OFA) use, in high-incidence MI subgroup | OFA use or not | 4 | 305 / 13,168 | RR | 0.52 | 0.027 | Yes | 33.7% | Yes | No | Low | Critically low |
| Zhao, 2009 | OFA use, in low-incidence MI subgroup | OFA use or not | 4 | 149 /  7,829 | RR | 1.39 (1.01, 1.92) | 0.045 | Yes | 0% | No | NP | Low | Critically low |
| Khoueiry, 2013 | OFA use, in patients with recent MI | OFA use or not | 5 | 286 / | OR | 0.69 | 0.003 | Yes | 0% | No | NP | Low | Critically low |
|  |  |  |  | 13,126 |  | (0.55, 0.88) |  |  |  |  |  |  |  |
| Leon, 2008 | Fish oil | Use versus no use | 6 | 338/31111 | OR | 0.81 (0.52, 1.25) | 0.343 | Yes | 23.2% | No | NP | NS | High quality |
| Leon, 2008 | Fish oil | Use versus no use in patients with coronary artery disease | 4 | 332/30509 | OR | 0.75 (0.53, 1.06) | 0.105 | Yes | 15.5% | No | NP | NS | High quality |
| Fernandes, 2019 | Trans-endocardial stem cell injections with skeletal myoblast | Use versus no use for chronic ischemic heart disease | 3 | 11/83 | OR | 2.50 (0.54, 11.58) | 0.242 | Yes | 0.0% | No | NP | NS | High quality |
| Fernandes, 2019 | Trans-endocardial stem cell injections (with other cells and skeletal myoblast) | Use versus no use for chronic ischemic heart disease | 7 | 25/505 | OR | 0.65 (0.22, 1.91) | 0.438 | Yes | 12.8% | No | NP | NS | High quality |
| **Non-ischemic Cardiomyopathy population** |  |  |  |  |  |  |  |  |  |  |  |  |  |
| Peck, 2014 | ACEi and beta-blocker use, in patients with LV dysfunction without recent MI | Use or not | 9 | 2,461 / 29,540 | RR | 0.91 (0.82, 1.00) | 0.050 | Yes | 29.9% | No | No | High | High |
| Kolodziejczak, 2017 | ICD use, in patients with non-ischemic heart disease (non-IHD) | ICD use or conventional therapy | 3 | 90 / 1,677 | HR | 0.44 (0.28, 0.69) | 3.41 x 10^-4^ | Yes | 0% | No | No | Moderate | High |
| Siddiqui, 2018 | ICD and cardiac resynchronization therapy with ICD (CRT-D), in patients with non-IHD | CRT-D or medical management | 3 | 90 / 1,677 | OR | 0.44 (0.28, 0.70) | 0.001 | Yes | 0% | No | No | Moderate | High |
| **Severe Aortic Stenosis population** | | | |  |  |  |  |  |  |  |  |  |  |
| Rafique, 2009 | stress test | Abnormal vs normal stress test | 3 | 9/237 | OR | 0.32 (0.05, 2.24) | 0.252 | Yes | 0.0% | No | NP | NS | Moderate quality |
| **Hypertension population** |  |  |  |  |  |  |  |  |  |  |  |  |  |
| Hebert, 2008 | Epithelial sodium channel inhibitors combined with a thiazide diuretic | Use or not | 3 | 100 /  5,761 | OR | 0.61 (0.39, 0.95) | 0.029 | Yes | 0% | No | NP | Low | Critically low |
| Taverny, 2016 | Antihypertensives | Use versus no use | 15 | 493/29905 | RR | 0.96 (0.81, 1.15) | 0.687 | Yes | 0.0% | No | NP | NS | High quality |
| Taverny, 2016 | First line diuretics | Use versus no use in patients with hypertension | 10 | 197/17894 | RR | 1.07 (0.81, 1.42) | 0.633 | Yes | 0.0% | No | NP | NS | High quality |
| Taverny, 2016 | First line other antihypertensive drugs | Use versus no use in patients with hypertension | 5 | 280/18369 | RR | 0.94 | 0.606 | Yes | 0.0% | No | NP | NS | High quality |
| Taverny, 2016 | Antihypertensives (First line diuretics and other antihypertensive drugs) | Use versus no use in patients with hypertension | 15 | 477/36263 | RR | 0.99 (0.83, 1.19) | 0.924 | Yes | 0.0% | No | NP | NS | High quality |
| Al-Gobari, 2018 | Angiotensin receptor blockers (ARBs) and ACEIs | Use versus no use in patients with hypertension | 3 | 864/8704 | RR | 1.01 (0.80, 1.28) | 0.916 | Yes | 57.5% | No | NP | NS | High quality |
| **High risk population for SCD** |  |  |  |  |  |  |  |  |  |  |  |  |  |
| Claro, 2015 | Amiodarone use, for primary prevention | Amiodarone use or not | 17 | 666/ 8,386 | RR | 0.76 (0.66, 0.88) | 1.98 x 10^-4^ | No | 0% | No | NP | Low | Critically low |
| Levantesi, 2007 | Statin use | Statin use or not | 10 | 688 / 22,275 | OR | 0.79 (0.67, 0.94) | 0.008 | Yes | 9.8% | No | No | Low | Critically low |
| Claro, 2015 | Amiodarone | amiodarone vs other antiarrhythmics | 3 | 25/540 | RR | 0.44 (0.19, 1.02) | 0.056 | Yes | 0.0% | No | NP | NS | Critically Low Quality |
| Claro, 2015 | Amiodarone | amiodarone vs b blockers | 2 | 13/342 | RR | 0.37 (0.11, 1.26) | 0.113 | NA | 0.0% | NA | NP | NS | Critically Low Quality |
| Claro, 2015 | Amiodarone | amiodarone vs placebo for secondary prevention | 2 | 8/440 | RR | 4.32 (0.87, 21.52) | 0.074 | NA | 0.0% | NA | NP | NS | Critically Low Quality |
| Claro, 2015 | Amiodarone | amiodarone vs other antiarrhythmics for secondary prevention | 4 | 102/839 | RR | 1.40 (0.56, 3.50) | 0.469 | Yes | 71.8% | No | No | NS | Critically Low Quality |
| Claro, 2015 | Amiodarone | amiodarone vs other antiarrhythmics for secondary prevention in patients without ICD | 2 | 65/234 | RR | 0.95 (0.46, 1.97) | 0.894 | NA | 14.3% | NA | NP | NS | Critically Low Quality |
| Khoueiry, 2013 | omega-3 fatty acids | Use versus no use omega-3 fatty acids have no effect on the incidence of sudden cardiac death in patients with history of ventricular arrhythmia and implantable cardioverter-defibrillator | 3 | 441/1148 | OR | 0.97 (0.57, 1.66) | 0.915 | Yes | 74.8% | No | No | NS | Critically Low Quality |
| **Prostate cancer population** | | |  |  |  |  |  |  |  |  |  |  |  |
| Liang, 2019 | androgen deprivation therapy | Use versus no use | 3 | 213/1716 | RR | 1.30 (0.68, 2.48) | 0.429 | Yes | 82.2% | No | No | NS | High quality |
| **Other categories** | |  |  |  |  |  |  |  |  |  |  |  |  |
| Chen, 2011 | OFA use, in non-guidelines-adjusted therapy subgroup | OFA use or not | 6 | 308 / 14,219 | RR | 0.67 (0.54, 0.84) | 0.001 | No | 0% | Yes | No | Moderate | Critically low |
| Skeaff, 2009 | fish or n–3 PUFA | Use versus no use | 6 | 1065/4086 | RR | 1.02 (0.78, 1.34) | 0.872 | Yes | 60.9% | No | Yes | NS | Moderate quality |
| Zhao, 2009 | omega-3 fatty acids | Use versus no use in high and low-incidence myocardial infarction subgroup | 8 | 454/20997 | RR | 0.76 (0.47, 1.23) | 0.260 | Yes | 63.5% | No | No | NS | Critically Low Quality |
| Chen, 2011 | omega-3 fatty acids | Use versus no use in guidelines-adjusted therapy subgroup | 4 | 772/19210 | RR | 0.96 (0.84, 1.09) | 0.517 | Yes | 0.0% | No | NP | NS | Critically Low Quality |
| Chen, 2011 | omega-3 fatty acids | Use versus no use in guidelines-adjusted therapy subgroup and in non-guidelines-adjusted therapy subgroup | 10 | 1080/33429 | RR | 0.84 (0.69, 1.02) | 0.084 | Yes | 28.2% | No | No | NS | Critically Low Quality |
| Khoueiry, 2013 | omega-3 fatty acids | Use versus no use | 9 | 743/32919 | OR | 0.85 (0.62, 1.17) | 0.328 | Yes | 51.7% | No | No | NS | Critically Low Quality |

Notes: CE, class of evidence; CI, confidence interval; ES, effect size; ESB, excess significance bias; GLE: GRADE level of evidence; GRADE: GRADE, Grading of Recommendations Assessment, Development and Evaluation; I2, heterogeneity; K, number of studies for each factor; LS, largest study with significant effect; n, number of cases; N, total number of cohort per factor; NA, not assessable; NR, not reported; NP, not pertinent because the number of observed studies is less than the expected; OR, odds ratio; RR, risk ratio; PI, prediction interval; SCD: sudden cardiac death; SSE, small study effects; sign., significant; eOR>1 indicates risk factor. BMI: Body Mass index, ACE: Angiotensin Converting enzyme; ARB: angiotensin receptor blocker BMI: Body mass index; CRT: cardiac resynchronization therapy; BP: blood pressure; ICD: Implantable cardioverter defibrillators LGE: Late gadolinium enhancement; MRA: mineralocorticoid receptor antagonist; MRI: magnetic resonance imaging; PVC: Premature Ventricular Complex; RCT: randomized controlled studies; SCD: sudden cardiac death; SD: standard deviation; SGLT-2: Sodium-glucose cotransporter-2 inhibitors (SGLT2)

**References**

1. Neumärker K-J. Mortality and sudden death in anorexia nervosa. *International Journal of Eating Disorders* 1997; 21: 205-212. DOI: <https://doi.org/10.1002/(SICI)1098-108X(199704)21:3><205::AID-EAT1>3.0.CO;2-O.

2. Sim I, McDonald KM, Lavori PW, et al. Quantitative overview of randomized trials of amiodarone to prevent sudden cardiac death. *Circulation* 1997; 96: 2823-2829. 1997/12/31. DOI: 10.1161/01.cir.96.9.2823.

3. Piccini JP, Berger JS and O'Connor CM. Amiodarone for the prevention of sudden cardiac death: a meta-analysis of randomized controlled trials. *Eur Heart J* 2009; 30: 1245-1253. 2009/04/02. DOI: 10.1093/eurheartj/ehp100.

4. Ottani F, Galvani M, Nicolini FA, et al. Elevated cardiac troponin levels predict the risk of adverse outcome in patients with acute coronary syndromes. *Am Heart J* 2000; 140: 917-927. 2000/12/02. DOI: 10.1067/mhj.2000.111107.

5. Pourati I, Hyder M and Rosenthal L. Indications for implantable cardiac defibrillators in patients with congestive heart failure: implications of the sudden cardiac death in heart failure trial. *Curr Cardiol Rep* 2005; 7: 223-228. 2005/05/04. DOI: 10.1007/s11886-005-0081-8.

6. Witte DR, Grobbee DE, Bots ML, et al. A meta-analysis of excess cardiac mortality on Monday. *Eur J Epidemiol* 2005; 20: 401-406. 2005/08/06. DOI: 10.1007/s10654-004-8783-6.

7. Lam SKH and Owen A. Does cardiac resynchronization therapy reduce sudden cardiac deaths? *European Heart Journal* 2007; 28: 1268-1268. DOI: 10.1093/eurheartj/ehm096.

8. Rahimi K, Majoni W, Merhi A, et al. Effect of statins on ventricular tachyarrhythmia, cardiac arrest, and sudden cardiac death: a meta-analysis of published and unpublished evidence from randomized trials. *European Heart Journal* 2012; 33: 1571-1581. DOI: 10.1093/eurheartj/ehs005.

9. Al-Gobari M, Le HH, Fall M, et al. No benefits of statins for sudden cardiac death prevention in patients with heart failure and reduced ejection fraction: A meta-analysis of randomized controlled trials. *PLoS One* 2017; 12: e0171168. 2017/02/07. DOI: 10.1371/journal.pone.0171168.

10. Alboni P, Favaron E, Paparella N, et al. Is there an association between depression and cardiovascular mortality or sudden death? *J Cardiovasc Med (Hagerstown)* 2008; 9: 356-362. 2008/03/13. DOI: 10.2459/JCM.0b013e3282785240.

11. de Sousa MR, Morillo CA, Rabelo FT, et al. Non-sustained ventricular tachycardia as a predictor of sudden cardiac death in patients with left ventricular dysfunction: a meta-analysis. *Eur J Heart Fail* 2008; 10: 1007-1014. 2008/08/12. DOI: 10.1016/j.ejheart.2008.07.002.

12. Scott PA, Barry J, Roberts PR, et al. Brain natriuretic peptide for the prediction of sudden cardiac death and ventricular arrhythmias: a meta-analysis. *European Journal of Heart Failure* 2009; 11: 958-966. DOI: <https://doi.org/10.1093/eurjhf/hfp123>.

13. Cao L, Zheng H, Xie Y, et al. [Effect of preoxygenation and apnoeic oxygenation during intubation in the critically ill patients: a network Meta-analysis]. *Zhonghua Wei Zhong Bing Ji Jiu Yi Xue* 2019; 31: 1236-1241. 2019/11/28. DOI: 10.3760/cma.j.issn.2095-4352.2019.10.011.

14. Christiaans I, van Engelen K, van Langen IM, et al. Risk stratification for sudden cardiac death in hypertrophic cardiomyopathy: systematic review of clinical risk markers. *Europace* 2010; 12: 313-321. 2010/02/02. DOI: 10.1093/europace/eup431.

15. Leonardi RA, Kransdorf EP, Simel DL, et al. Meta-analyses of septal reduction therapies for obstructive hypertrophic cardiomyopathy: comparative rates of overall mortality and sudden cardiac death after treatment. *Circ Cardiovasc Interv* 2010; 3: 97-104. 2010/03/04. DOI: 10.1161/circinterventions.109.916676.

16. Sealy DP, Pekarek L, Russ D, et al. Vital signs and demographics in the preparticipation sports exam: do they help us find the elusive athlete at risk for sudden cardiac death? *Curr Sports Med Rep* 2010; 9: 338-341. 2010/11/12. DOI: 10.1249/JSR.0b013e3182014ed6.

17. Garritano NF and Willmarth-Stec M. Student athletes, sudden cardiac death, and lifesaving legislation: a review of the literature. *J Pediatr Health Care* 2015; 29: 233-242. 2015/01/13. DOI: 10.1016/j.pedhc.2014.11.006.

18. Gavin MC, Newton-Cheh C, Gaziano JM, et al. A common variant in the β2-adrenergic receptor and risk of sudden cardiac death. *Heart Rhythm* 2011; 8: 704-710. 2011/01/11. DOI: 10.1016/j.hrthm.2011.01.003.

19. Musa-Veloso K, Binns MA, Kocenas A, et al. Impact of low v. moderate intakes of long-chain n-3 fatty acids on risk of coronary heart disease. *British Journal of Nutrition* 2011; 106: 1129-1141. 2011/05/31. DOI: 10.1017/S0007114511001644.

20. Lahtinen AM, Noseworthy PA, Havulinna AS, et al. Common genetic variants associated with sudden cardiac death: the FinSCDgen study. *PLoS One* 2012; 7: e41675. 2012/07/31. DOI: 10.1371/journal.pone.0041675.

21. Rodday AM, Triedman JK, Alexander ME, et al. Electrocardiogram screening for disorders that cause sudden cardiac death in asymptomatic children: a meta-analysis. *Pediatrics* 2012; 129: e999-1010. 2012/03/07. DOI: 10.1542/peds.2011-0643.

22. Ahn J, Kim HJ, Choi JI, et al. Effectiveness of beta-blockers depending on the genotype of congenital long-QT syndrome: A meta-analysis. *PLoS One* 2017; 12: e0185680. 2017/10/24. DOI: 10.1371/journal.pone.0185680.

23. Rossello X, Ariti C, Pocock SJ, et al. Impact of mineralocorticoid receptor antagonists on the risk of sudden cardiac death in patients with heart failure and left-ventricular systolic dysfunction: an individual patient-level meta-analysis of three randomized-controlled trials. *Clin Res Cardiol* 2019; 108: 477-486. 2018/09/29. DOI: 10.1007/s00392-018-1378-0.

24. Bezzina CR, Barc J, Mizusawa Y, et al. Common variants at SCN5A-SCN10A and HEY2 are associated with Brugada syndrome, a rare disease with high risk of sudden cardiac death. *Nat Genet* 2013; 45: 1044-1049. 2013/07/23. DOI: 10.1038/ng.2712.

25. Delise P, Probst V, Allocca G, et al. Clinical outcome of patients with the Brugada type 1 electrocardiogram without prophylactic implantable cardioverter defibrillator in primary prevention: a cumulative analysis of seven large prospective studies. *EP Europace* 2017; 20: f77-f85. DOI: 10.1093/europace/eux226.

26. Chen LY, Sotoodehnia N, Bůžková P, et al. Atrial fibrillation and the risk of sudden cardiac death: the atherosclerosis risk in communities study and cardiovascular health study. *JAMA Intern Med* 2013; 173: 29-35. 2013/02/14. DOI: 10.1001/2013.jamainternmed.744.

27. Chen Z, Shi Y, Hou X, et al. Microvolt T-wave alternans for risk stratification of cardiac events in ischemic cardiomyopathy: A meta-analysis. *International Journal of Cardiology* 2013; 167: 2061-2065. DOI: <https://doi.org/10.1016/j.ijcard.2012.05.050>.

28. Liebregts M, Vriesendorp PA, Mahmoodi BK, et al. A Systematic Review and Meta-Analysis of Long-Term Outcomes After Septal Reduction Therapy in Patients With Hypertrophic Cardiomyopathy. *JACC Heart Fail* 2015; 3: 896-905. 2015/10/12. DOI: 10.1016/j.jchf.2015.06.011.

29. Ullal AJ, Abdelfattah RS, Ashley EA, et al. Hypertrophic Cardiomyopathy as a Cause of Sudden Cardiac Death in the Young: A Meta-Analysis. *The American journal of medicine* 2016; 129: 486-496.e482. 2016/01/24. DOI: 10.1016/j.amjmed.2015.12.027.

30. Bittencourt MI, Cader SA, Araújo DV, et al. Role of Myocardial Fibrosis in Hypertrophic Cardiomyopathy: A Systematic Review and Updated Meta-Analysis of Risk Markers for Sudden Death. *Arquivos Brasileiros de Cardiologia* 2019; 112: 281-289.

31. O'Mahony C, Akhtar MM, Anastasiou Z, et al. Effectiveness of the 2014 European Society of Cardiology guideline on sudden cardiac death in hypertrophic cardiomyopathy: a systematic review and meta-analysis. *Heart* 2019; 105: 623-631. 2018/10/28. DOI: 10.1136/heartjnl-2018-313700.

32. Wang JP, Zhang YM, Yang RJ, et al. Efficacy and safety of active abdominal compression-decompression versus standard CPR for cardiac arrests: A systematic review and meta-analysis of 17 RCTs. *Int J Surg* 2019; 71: 132-139. 2019/09/29. DOI: 10.1016/j.ijsu.2019.09.026.

33. Zeitler EP and Sun AY. The J wave patterns and risk of sudden cardiac death in patients with coronary artery disease. *J Electrocardiol* 2013; 46: 446-450. 2013/08/29. DOI: 10.1016/j.jelectrocard.2013.06.026.

34. Goldberger JJ, Subačius H, Patel T, et al. Sudden cardiac death risk stratification in patients with nonischemic dilated cardiomyopathy. *J Am Coll Cardiol* 2014; 63: 1879-1889. 2014/01/22. DOI: 10.1016/j.jacc.2013.12.021.

35. Lemaitre RN, Johnson CO, Hesselson S, et al. Common variation in fatty acid metabolic genes and risk of incident sudden cardiac arrest. *Heart Rhythm* 2014; 11: 471-477. 2014/01/15. DOI: 10.1016/j.hrthm.2014.01.008.

36. Pun PH, Al-Khatib SM, Han JY, et al. Implantable cardioverter-defibrillators for primary prevention of sudden cardiac death in CKD: a meta-analysis of patient-level data from 3 randomized trials. *American journal of kidney diseases : the official journal of the National Kidney Foundation* 2014; 64: 32-39. 2014/02/13. DOI: 10.1053/j.ajkd.2013.12.009.

37. Zaccardi F, Khan H and Laukkanen JA. Diabetes mellitus and risk of sudden cardiac death: a systematic review and meta-analysis. *Int J Cardiol* 2014; 177: 535-537. 2014/09/06. DOI: 10.1016/j.ijcard.2014.08.105.

38. Hernesniemi JA, Lyytikäinen LP, Oksala N, et al. Predicting sudden cardiac death using common genetic risk variants for coronary artery disease. *Eur Heart J* 2015; 36: 1669-1675. 2015/04/25. DOI: 10.1093/eurheartj/ehv106.

39. Kunutsor SK, Kurl S, Zaccardi F, et al. Baseline and long-term fibrinogen levels and risk of sudden cardiac death: A new prospective study and meta-analysis. *Atherosclerosis* 2016; 245: 171-180. 2016/01/03. DOI: 10.1016/j.atherosclerosis.2015.12.020.

40. Ramesh S, Zalucky A, Hemmelgarn BR, et al. Incidence of sudden cardiac death in adults with end-stage renal disease: a systematic review and meta-analysis. *BMC Nephrology* 2016; 17: 78. DOI: 10.1186/s12882-016-0293-8.

41. Weng Z, Yao J, Chan RH, et al. Prognostic Value of LGE-CMR in HCM: A Meta-Analysis. *JACC Cardiovasc Imaging* 2016; 9: 1392-1402. 2016/07/28. DOI: 10.1016/j.jcmg.2016.02.031.

42. Zhang X, Shen C, Zhai S, et al. A meta-analysis of the effects of β-adrenergic blockers in chronic heart failure. *Exp Ther Med* 2016; 12: 2489-2496. 2016/10/06. DOI: 10.3892/etm.2016.3657.

43. Akel T and Lafferty J. Implantable cardioverter defibrillators for primary prevention in patients with nonischemic cardiomyopathy: A systematic review and meta-analysis. *Cardiovascular Therapeutics* 2017; 35: e12253. DOI: <https://doi.org/10.1111/1755-5922.12253>.

44. Al-Khatib SM, Fonarow GC, Joglar JA, et al. Primary Prevention Implantable Cardioverter Defibrillators in Patients With Nonischemic Cardiomyopathy: A Meta-analysis. *JAMA Cardiol* 2017; 2: 685-688. 2017/03/30. DOI: 10.1001/jamacardio.2017.0630.

45. Anantha Narayanan M, Vakil K, Reddy YN, et al. Efficacy of Implantable Cardioverter-Defibrillator Therapy in Patients With Nonischemic Cardiomyopathy: A Systematic Review and Meta-Analysis of Randomized Controlled Trials. *JACC Clin Electrophysiol* 2017; 3: 962-970. 2018/05/16. DOI: 10.1016/j.jacep.2017.02.006.

46. Elayi CS, Charnigo RJ, Heron PM, et al. Primary Prevention of Sudden Cardiac Death Early Post-Myocardial Infarction: Root Cause Analysis for Implantable Cardioverter-Defibrillator Failure and Currently Available Options. *Circ Arrhythm Electrophysiol* 2017; 10 2017/06/21. DOI: 10.1161/circep.117.005194.

47. Di Marco A, Anguera I, Schmitt M, et al. Late Gadolinium Enhancement and the Risk for Ventricular Arrhythmias or Sudden Death in Dilated Cardiomyopathy: Systematic Review and Meta-Analysis. *JACC Heart Fail* 2017; 5: 28-38. 2016/12/27. DOI: 10.1016/j.jchf.2016.09.017.

48. Tereshchenko LG, Soliman EZ, Davis BR, et al. Risk stratification of sudden cardiac death in hypertension. *J Electrocardiol* 2017; 50: 798-801. 2017/09/17. DOI: 10.1016/j.jelectrocard.2017.08.012.

49. Xing Z, Tang L, Chen C, et al. Effectiveness of Implantation of Cardioverter-Defibrillators Therapy in Patients with Non-Ischemic Heart Failure: an Updated Systematic Review and Meta-Analysis. *Braz J Cardiovasc Surg* 2017; 32: 417-422. 2017/12/07. DOI: 10.21470/1678-9741-2017-0003.

50. Alba AC, Foroutan F, Ng Fat Hing NKV, et al. Incidence and predictors of sudden cardiac death after heart transplantation: A systematic review and meta-analysis. *Clinical Transplantation* 2018; 32: e13206. DOI: <https://doi.org/10.1111/ctr.13206>.

51. Alba AC, Foroutan F, Duero Posada J, et al. Implantable cardiac defibrillator and mortality in non-ischaemic cardiomyopathy: an updated meta-analysis. *Heart* 2018; 104: 230-236. 2017/08/07. DOI: 10.1136/heartjnl-2017-311430.

52. Ashar FN, Mitchell RN, Albert CM, et al. A comprehensive evaluation of the genetic architecture of sudden cardiac arrest. *Eur Heart J* 2018; 39: 3961-3969. 2018/09/01. DOI: 10.1093/eurheartj/ehy474.

53. Liu X, Shi J and Xiao P. Associations between common ion channel single nucleotide polymorphisms and sudden cardiac death in adults: A MOOSE-compliant meta-analysis. *Medicine (Baltimore)* 2018; 97: e12428. 2018/09/22. DOI: 10.1097/md.0000000000012428.

54. Barra S, Providência R, Tang A, et al. Importance of Implantable Cardioverter-Defibrillator Back-Up in Cardiac Resynchronization Therapy Recipients: A Systematic Review and Meta-Analysis. *J Am Heart Assoc* 2015; 4 2015/11/08. DOI: 10.1161/jaha.115.002539.

55. Beggs SAS, Jhund PS, Jackson CE, et al. Non-ischaemic cardiomyopathy, sudden death and implantable defibrillators: a review and meta-analysis. *Heart* 2018; 104: 144-150. 2017/10/08. DOI: 10.1136/heartjnl-2016-310850.

56. Bor S, Demir M, Ozdemir O, et al. A meta-analysis on the cardiac safety profile of domperidone compared to metoclopramide. *United European Gastroenterol J* 2018; 6: 1331-1346. 2018/11/06. DOI: 10.1177/2050640618799153.

57. Cai JZ, Zhu YX, Wang XY, et al. Comparison of new-generation drug-eluting stents versus drug-coated balloon for in-stent restenosis: a meta-analysis of randomised controlled trials. *BMJ Open* 2018; 8: e017231. 2018/02/24. DOI: 10.1136/bmjopen-2017-017231.

58. Simoons M, Seldenrijk A, Mulder H, et al. Limited Evidence for Risk Factors for Proarrhythmia and Sudden Cardiac Death in Patients Using Antidepressants: Dutch Consensus on ECG Monitoring. *Drug Saf* 2018; 41: 655-664. 2018/02/28. DOI: 10.1007/s40264-018-0649-z.

59. Cadrin-Tourigny J, Bosman LP, Tadros R, et al. Risk stratification for ventricular arrhythmias and sudden cardiac death in arrhythmogenic right ventricular cardiomyopathy: an update. *Expert Rev Cardiovasc Ther* 2019; 17: 645-651. 2019/08/20. DOI: 10.1080/14779072.2019.1657831.

60. Nalliah CJ, Mahajan R, Elliott AD, et al. Mitral valve prolapse and sudden cardiac death: a systematic review and meta-analysis. *Heart* 2019; 105: 144-151. 2018/09/23. DOI: 10.1136/heartjnl-2017-312932.

61. Kerpen K, Koutrolou-Sotiropoulou P, Zhu C, et al. Disparities in death rates in women with peripartum cardiomyopathy between advanced and developing countries: A systematic review and meta-analysis. *Arch Cardiovasc Dis* 2019; 112: 187-198. 2018/12/31. DOI: 10.1016/j.acvd.2018.10.002.

62. Lalande E, Burwash-Brennan T, Burns K, et al. Is point-of-care ultrasound a reliable predictor of outcome during atraumatic, non-shockable cardiac arrest? A systematic review and meta-analysis from the SHoC investigators. *Resuscitation* 2019; 139: 159-166. 2019/04/12. DOI: 10.1016/j.resuscitation.2019.03.027.

63. Napp LC. The Risk of Takotsubo Syndrome: Seeing the Light. *JACC Heart Fail* 2019; 7: 155-157. 2019/01/07. DOI: 10.1016/j.jchf.2018.11.012.

64. Moe SM, Long J, Schwantes-An TL, et al. Angiotensin-related genetic determinants of cardiovascular disease in patients undergoing hemodialysis. *Nephrol Dial Transplant* 2019; 34: 1924-1931. 2018/07/10. DOI: 10.1093/ndt/gfy191.

65. Osman M, Kheiri B, Osman K, et al. Alcohol septal ablation vs myectomy for symptomatic hypertrophic obstructive cardiomyopathy: Systematic review and meta-analysis. *Clinical Cardiology* 2019; 42: 190-197. DOI: <https://doi.org/10.1002/clc.23113>.

66. Bazoukis G, Yeung C, Wui Hang Ho R, et al. Association of QT dispersion with mortality and arrhythmic events-A meta-analysis of observational studies. *J Arrhythm* 2020; 36: 105-115. 2020/02/20. DOI: 10.1002/joa3.12253.

67. Aidelsburger P, Seyed-Ghaemi J, Guinin C, et al. Effectiveness, efficacy, and safety of wearable cardioverter-defibrillators in the treatment of sudden cardiac arrest - Results from a health technology assessment. *Int J Technol Assess Health Care* 2020: 1-9. 2020/07/01. DOI: 10.1017/s0266462320000379.

68. Cao Z, Zhao M, Xu C, et al. Evaluation of Agonal Cardiac Function for Sudden Cardiac Death in Forensic Medicine with Postmortem Brain Natriuretic Peptide (BNP) and NT-proBNP: A Meta-analysis. *Journal of Forensic Sciences* 2020; 65: 686-691. DOI: <https://doi.org/10.1111/1556-4029.14232>.

69. Chahal CAA, Salloum MN, Alahdab F, et al. Systematic Review of the Genetics of Sudden Unexpected Death in Epilepsy: Potential Overlap With Sudden Cardiac Death and Arrhythmia-Related Genes. *J Am Heart Assoc* 2020; 9: e012264. 2019/12/24. DOI: 10.1161/jaha.119.012264.

70. Heilbrunn E, Ssentongo P, Chinchilli VM, et al. Sudden death in individuals with obstructive sleep apnoea: protocol for a systematic review and meta-analysis. *BMJ Open* 2020; 10: e039774. 2020/08/28. DOI: 10.1136/bmjopen-2020-039774.

71. Lombardi M, Chiabrando JG, Vescovo GM, et al. Impact of Different Doses of Omega-3 Fatty Acids on Cardiovascular Outcomes: a Pairwise and Network Meta-analysis. *Curr Atheroscler Rep* 2020; 22: 45. 2020/07/17. DOI: 10.1007/s11883-020-00865-5.

72. Possner M, Tseng SY, Alahdab F, et al. Risk Factors for Mortality and Ventricular Tachycardia in Patients With Repaired Tetralogy of Fallot: A Systematic Review and Meta-analysis. *Can J Cardiol* 2020; 36: 1815-1825. 2020/05/18. DOI: 10.1016/j.cjca.2020.01.023.

73. Zhu Y, Huang H, Feng J, et al. Therapeutic hypothermia for cardiac arrest due to non-shockable rhythm: A protocol for systematic review and meta-analysis. *Medicine (Baltimore)* 2020; 99: e21452. 2020/09/03. DOI: 10.1097/md.0000000000021452.

74. Yu Y, Meng Q, Munot S, et al. Assessment of Community Interventions for Bystander Cardiopulmonary Resuscitation in Out-of-Hospital Cardiac Arrest: A Systematic Review and Meta-analysis. *JAMA Network Open* 2020; 3: e209256-e209256. DOI: 10.1001/jamanetworkopen.2020.9256.

75. Yao RQ, Xia DM, Wang LX, et al. Clinical Efficiency of Vasopressin or Its Analogs in Comparison With Catecholamines Alone on Patients With Septic Shock: A Systematic Review and Meta-Analysis. *Front Pharmacol* 2020; 11: 563. 2020/05/22. DOI: 10.3389/fphar.2020.00563.

76. Turley TN, O’Byrne MM, Kosel ML, et al. Identification of Susceptibility Loci for Spontaneous Coronary Artery Dissection. *JAMA Cardiology* 2020; 5: 929-938. DOI: 10.1001/jamacardio.2020.0872.

77. Yan S, Gan Y, Jiang N, et al. The global survival rate among adult out-of-hospital cardiac arrest patients who received cardiopulmonary resuscitation: a systematic review and meta-analysis. *Critical care* 2020; 24: 61. 2020/02/24. DOI: 10.1186/s13054-020-2773-2.

78. Wilson ME, Mittal A, Karki B, et al. Do-not-intubate orders in patients with acute respiratory failure: a systematic review and meta-analysis. *Intensive Care Med* 2020; 46: 36-45. 2019/10/30. DOI: 10.1007/s00134-019-05828-2.

79. Ullah W, Gowda SN and Fischman D. Safety and Efficacy of Colchicine in Patients With Coronary Artery Disease: A Systematic Review and Meta-Analysis. *Cardiovasc Revasc Med* 2021; 23: 1-6. 2020/06/20. DOI: 10.1016/j.carrev.2020.06.004.

80. Turner JS, Bucca AW, Propst SL, et al. Association of Checklist Use in Endotracheal Intubation With Clinically Important Outcomes: A Systematic Review and Meta-analysis. *JAMA Netw Open* 2020; 3: e209278. 2020/07/03. DOI: 10.1001/jamanetworkopen.2020.9278.

81. Tse G, Lee S, Gong M, et al. Restitution metrics in Brugada syndrome: a systematic review and meta-analysis. *Journal of interventional cardiac electrophysiology : an international journal of arrhythmias and pacing* 2020; 57: 319-327. 2019/12/15. DOI: 10.1007/s10840-019-00675-z.

82. Tran A, Fernando SM, Rochwerg B, et al. Pre-arrest and intra-arrest prognostic factors associated with survival following traumatic out-of-hospital cardiac arrest - A systematic review and meta-analysis. *Resuscitation* 2020; 153: 119-135. 2020/06/13. DOI: 10.1016/j.resuscitation.2020.05.052.

83. Tian C, An N, Yuan M, et al. A Pooled Analysis of the Prognostic Significance of Brugada Syndrome with Atrial Fibrillation. *Curr Pharm Des* 2020; 26: 129-137. 2020/01/17. DOI: 10.2174/1381612826666200114112029.

84. Takagi H, Hari Y, Nakashima K, et al. Meta-analysis of propensity matched studies of robotic versus conventional mitral valve surgery. *Journal of cardiology* 2020; 75: 177-181. 2019/08/25. DOI: 10.1016/j.jjcc.2019.06.014.

85. Spirito A, Gargiulo G, Siontis GCM, et al. Cardiovascular mortality and morbidity in patients undergoing percutaneous coronary intervention after out-of-hospital cardiac arrest: a systematic review and meta-analysis. *EuroIntervention* 2021; 16: e1245-e1253. 2020/07/07. DOI: 10.4244/eij-d-20-00221.

86. Sammani A, Kayvanpour E, Bosman LP, et al. Predicting sustained ventricular arrhythmias in dilated cardiomyopathy: a meta-analysis and systematic review. *ESC Heart Failure* 2020; 7: 1430-1441. DOI: <https://doi.org/10.1002/ehf2.12689>.

87. Sahu AK, Bhoi S, Aggarwal P, et al. Endotracheal Tube Placement Confirmation by Ultrasonography: A Systematic Review and Meta-Analysis of more than 2500 Patients. *The Journal of Emergency Medicine* 2020; 59: 254-264. DOI: <https://doi.org/10.1016/j.jemermed.2020.04.040>.

88. Rout A, Singh S, Sarkar S, et al. Meta-Analysis of the Usefulness of Therapeutic Hypothermia After Cardiac Arrest. *The American Journal of Cardiology* 2020; 133: 48-53. DOI: <https://doi.org/10.1016/j.amjcard.2020.07.038>.

89. Pranata R, Yonas E, Vania R, et al. Electrocardiographic early repolarization is associated with future ventricular arrhythmia after acute myocardial infarction—Systematic Review and Meta-Analysis. *Journal of Arrhythmia* 2019; 35: 626-635. DOI: <https://doi.org/10.1002/joa3.12196>.

90. Rassi FM, Minohara L, Rassi A, Jr., et al. Systematic Review and Meta-Analysis of Clinical Outcome After Implantable Cardioverter-Defibrillator Therapy in Patients With Chagas Heart Disease. *JACC Clin Electrophysiol* 2019; 5: 1213-1223. 2019/10/28. DOI: 10.1016/j.jacep.2019.07.003.

91. Rattanawong P, Chenbhanich J, Mekraksakit P, et al. SCN5A mutation status increases the risk of major arrhythmic events in Asian populations with Brugada syndrome: systematic review and meta-analysis. *Annals of Noninvasive Electrocardiology* 2019; 24: e12589. DOI: <https://doi.org/10.1111/anec.12589>.

92. Roterberg G, El-Battrawy I, Veith M, et al. Arrhythmic events in Brugada syndrome patients induced by fever. *Annals of Noninvasive Electrocardiology* 2020; 25: e12723. DOI: <https://doi.org/10.1111/anec.12723>.

93. Vlad C-E, Foia L, Popescu R, et al. Apolipoproteins A and B and PCSK9: Nontraditional Cardiovascular Risk Factors in Chronic Kidney Disease and in End-Stage Renal Disease. *Journal of Diabetes Research* 2019; 2019: 6906278. DOI: 10.1155/2019/6906278.

94. Yang Y, Hu D, Sacher F, et al. Meta-Analysis of Risk Stratification of SCN5A With Brugada Syndrome: Is SCN5A Always a Marker of Low Risk? *Front Physiol* 2019; 10: 103. 2019/03/07. DOI: 10.3389/fphys.2019.00103.

95. Zang X, Li S, Zhao Y, et al. Systematic Meta-Analysis of the Association Between a Common NOS1AP Genetic Polymorphism, the QTc Interval, and Sudden Death. *Int Heart J* 2019; 60: 1083-1090. 2019/08/27. DOI: 10.1536/ihj.19-024.

96. Mustafa U, Dherange P, Reddy R, et al. Atrial Fibrillation Is Associated With Higher Overall Mortality in Patients With Implantable Cardioverter&#x2010;Defibrillator: A Systematic Review and Meta&#x2010;Analysis. *Journal of the American Heart Association* 2018; 7: e010156. DOI: doi:10.1161/JAHA.118.010156.

97. Pickering JW, Blunt IRH and Than MP. Acute Kidney Injury and mortality prognosis in Acute Coronary Syndrome patients: A meta-analysis. *Nephrology* 2018; 23: 237-246. DOI: <https://doi.org/10.1111/nep.12984>.

98. Prins BP, Mead TJ, Brody JA, et al. Exome-chip meta-analysis identifies novel loci associated with cardiac conduction, including ADAMTS6. *Genome Biology* 2018; 19: 87. DOI: 10.1186/s13059-018-1457-6.

99. Rattanawong P, Riangwiwat T, Kanitsoraphan C, et al. Baseline fragmented QRS increases the risk of major arrhythmic events in hypertrophic cardiomyopathy: Systematic review and meta-analysis. *Annals of Noninvasive Electrocardiology* 2018; 23: e12533. DOI: <https://doi.org/10.1111/anec.12533>.

100. Shi S, Barajas-Martinez H, Liu T, et al. Prevalence of spontaneous Brugada ECG pattern recorded at standard intercostal leads: A meta-analysis. *Int J Cardiol* 2018; 254: 151-156. 2017/12/12. DOI: 10.1016/j.ijcard.2017.11.113.

101. Sunderland N, Kaura A, Murgatroyd F, et al. Outcomes with single-coil versus dual-coil implantable cardioverter defibrillators: a meta-analysis. *EP Europace* 2017; 20: e21-e29. DOI: 10.1093/europace/euw438.

102. Tse G, Gong M, Li CKH, et al. Tpeak-Tend, Tpeak-Tend/QT ratio and Tpeak-Tend dispersion for risk stratification in Brugada Syndrome: A systematic review and meta-analysis. *Journal of Arrhythmia* 2018; 34: 587-597. DOI: <https://doi.org/10.1002/joa3.12118>.

103. Zeitler EP, Sanders GD, Singh K, et al. Single vs. dual chamber implantable cardioverter-defibrillators or programming of implantable cardioverter-defibrillators in patients without a bradycardia pacing indication: systematic review and meta-analysis. *EP Europace* 2018; 20: 1621-1629. DOI: 10.1093/europace/euy183.

104. Brown PF, Miller C, Di Marco A, et al. Towards cardiac MRI based risk stratification in idiopathic dilated cardiomyopathy. *Heart* 2019; 105: 270-275. 2018/11/01. DOI: 10.1136/heartjnl-2018-313767.

105. Duma A, Maleczek M, Panjikaran B, et al. Major Adverse Cardiac Events and Mortality Associated with Electroconvulsive Therapy: A Systematic Review and Meta-analysis. *Anesthesiology* 2019; 130: 83-91. 2018/12/18. DOI: 10.1097/aln.0000000000002488.

106. Lei H, Hu J, Liu L, et al. Sex differences in survival after out-of-hospital cardiac arrest: a meta-analysis. *Critical care* 2020; 24: 613. 2020/10/21. DOI: 10.1186/s13054-020-03331-5.

107. Kawakami H, Nerlekar N, Haugaa KH, et al. Prediction of Ventricular Arrhythmias With Left Ventricular Mechanical Dispersion: A Systematic Review and Meta-Analysis. *JACC Cardiovasc Imaging* 2020; 13: 562-572. 2019/06/17. DOI: 10.1016/j.jcmg.2019.03.025.

108. Singh S, Fong HK, Mercedes BR, et al. COVID-19 and out-of-hospital cardiac arrest: A systematic review and meta-analysis. *Resuscitation* 2020; 156: 164-166. 2020/09/19. DOI: 10.1016/j.resuscitation.2020.08.133.

109. Hall KK, Lim A and Gale B. The Use of Rapid Response Teams to Reduce Failure to Rescue Events: A Systematic Review. *J Patient Saf* 2020; 16: S3-s7. 2020/08/19. DOI: 10.1097/pts.0000000000000748.

110. Couper K, Putt O, Field R, et al. Incidence of sudden cardiac death in the young: a systematic review. *BMJ Open* 2020; 10: e040815. 2020/10/10. DOI: 10.1136/bmjopen-2020-040815.

111. Rattanawong P, Kewcharoen J, Kanitsoraphan C, et al. The utility of drug challenge testing in Brugada syndrome: A systematic review and meta-analysis. *J Cardiovasc Electrophysiol* 2020; 31: 2474-2483. 2020/06/24. DOI: 10.1111/jce.14631.

112. Toloui A, Moshrefiaraghi D, Madani Neishaboori A, et al. Cardiac Complications and Pertaining Mortality Rate in COVID-19 Patients; a Systematic Review and Meta-Analysis. *Arch Acad Emerg Med* 2021; 9: e18. 2021/04/20. DOI: 10.22037/aaem.v9i1.1071.

113. Claro JC, Candia R, Rada G, et al. Amiodarone versus other pharmacological interventions for prevention of sudden cardiac death. *Cochrane Database of Systematic Reviews* 2015. DOI: 10.1002/14651858.CD008093.pub2.

114. Domanski MJ, Exner DV, Borkowf CB, et al. Effect of angiotensin converting enzyme inhibition on sudden cardiac death in patients following acute myocardial infarction. A meta-analysis of randomized clinical trials. *J Am Coll Cardiol* 1999; 33: 598-604. 1999/03/18. DOI: 10.1016/s0735-1097(98)00609-3.

115. Rivero-Ayerza M, Theuns DA, Garcia-Garcia HM, et al. Effects of cardiac resynchronization therapy on overall mortality and mode of death: a meta-analysis of randomized controlled trials. *Eur Heart J* 2006; 27: 2682-2688. 2006/09/13. DOI: 10.1093/eurheartj/ehl203.

116. Levantesi G, Scarano M, Marfisi R, et al. Meta-analysis of effect of statin treatment on risk of sudden death. *Am J Cardiol* 2007; 100: 1644-1650. 2007/11/27. DOI: 10.1016/j.amjcard.2007.07.015.

117. Shi S, Liu T, Liang J, et al. Depression and Risk of Sudden Cardiac Death and Arrhythmias: A Meta-Analysis. *Psychosom Med* 2017; 79: 153-161. 2016/09/15. DOI: 10.1097/psy.0000000000000382.

118. León H, Shibata MC, Sivakumaran S, et al. Effect of fish oil on arrhythmias and mortality: systematic review. *Bmj* 2008; 337: a2931. DOI: 10.1136/bmj.a2931.

119. Rafique AM, Biner S, Ray I, et al. Meta-analysis of prognostic value of stress testing in patients with asymptomatic severe aortic stenosis. *Am J Cardiol* 2009; 104: 972-977. 2009/09/22. DOI: 10.1016/j.amjcard.2009.05.044.

120. Skeaff CM and Miller J. Dietary fat and coronary heart disease: summary of evidence from prospective cohort and randomised controlled trials. *Ann Nutr Metab* 2009; 55: 173-201. 2009/09/16. DOI: 10.1159/000229002.

121. Zhao Y-T, Chen Q, Sun Y-X, et al. Prevention of sudden cardiac death with omega-3 fatty acids in patients with coronary heart disease: A meta-analysis of randomized controlled trials. *Annals of medicine* 2009; 41: 301-310. DOI: 10.1080/07853890802698834.

122. Chen Q, Cheng LQ, Xiao TH, et al. Effects of omega-3 fatty acid for sudden cardiac death prevention in patients with cardiovascular disease: a contemporary meta-analysis of randomized, controlled trials. *Cardiovasc Drugs Ther* 2011; 25: 259-265. 2011/06/01. DOI: 10.1007/s10557-011-6306-8.

123. Green JJ, Berger JS, Kramer CM, et al. Prognostic value of late gadolinium enhancement in clinical outcomes for hypertrophic cardiomyopathy. *JACC Cardiovasc Imaging* 2012; 5: 370-377. 2012/04/14. DOI: 10.1016/j.jcmg.2011.11.021.

124. Al-Gobari M, Khatib CE, Pillon F, et al. Beta-blockers for the prevention of sudden cardiac death in heart failure patients: a meta-analysis of randomized controlled trials. *BMC cardiovascular disorders* 2013; 13: 52. DOI: 10.1186/1471-2261-13-52.

125. Chatterjee S, Udell JA, Sardar P, et al. Comparable benefit of β-blocker therapy in heart failure across regions of the world: meta-analysis of randomized clinical trials. *Can J Cardiol* 2014; 30: 898-903. 2014/06/19. DOI: 10.1016/j.cjca.2014.03.012.

126. Khoueiry G, Abi Rafeh N, Sullivan E, et al. Do omega-3 polyunsaturated fatty acids reduce risk of sudden cardiac death and ventricular arrhythmias? A meta-analysis of randomized trials. *Heart & lung : the journal of critical care* 2013; 42: 251-256. 2013/05/30. DOI: 10.1016/j.hrtlng.2013.03.006.

127. Ataklte F, Erqou S, Laukkanen J, et al. Meta-analysis of ventricular premature complexes and their relation to cardiac mortality in general populations. *Am J Cardiol* 2013; 112: 1263-1270. 2013/08/10. DOI: 10.1016/j.amjcard.2013.05.065.

128. Bapoje SR, Bahia A, Hokanson JE, et al. Effects of mineralocorticoid receptor antagonists on the risk of sudden cardiac death in patients with left ventricular systolic dysfunction: a meta-analysis of randomized controlled trials. *Circ Heart Fail* 2013; 6: 166-173. 2013/02/14. DOI: 10.1161/circheartfailure.112.000003.

129. Barra S, Providência R, Duehmke R, et al. Cause-of-death analysis in patients with cardiac resynchronization therapy with or without a defibrillator: a systematic review and proportional meta-analysis. *EP Europace* 2017; 20: 481-491. DOI: 10.1093/europace/eux094.

130. Hebert PR, Coffey CS, Byrne DW, et al. Treatment of elderly hypertensive patients with epithelial sodium channel inhibitors combined with a thiazide diuretic reduces coronary mortality and sudden cardiac death. *J Am Soc Hypertens* 2008; 2: 355-365. 2009/09/04. DOI: 10.1016/j.jash.2008.04.001.

131. Calò L, De Santo T, Nuccio F, et al. Predictive value of microvolt T-wave alternans for cardiac death or ventricular tachyarrhythmic events in ischemic and nonischemic cardiomyopathy patients: a meta-analysis. *Annals of noninvasive electrocardiology : the official journal of the International Society for Holter and Noninvasive Electrocardiology, Inc* 2011; 16: 388-402. 2011/10/20. DOI: 10.1111/j.1542-474X.2011.00467.x.

132. Dahabreh IJ and Paulus JK. Association of Episodic Physical and Sexual Activity With Triggering of Acute Cardiac Events: Systematic Review and Meta-analysis. *JAMA : the journal of the American Medical Association* 2011; 305: 1225-1233. DOI: 10.1001/jama.2011.336.

133. Wu W, Tian L, Ke J, et al. Risk factors for cardiac events in patients with Brugada syndrome: A PRISMA-compliant meta-analysis and systematic review. *Medicine (Baltimore)* 2016; 95: e4214. 2016/07/30. DOI: 10.1097/md.0000000000004214.

134. Rattanawong P, Upala S, Riangwiwat T, et al. Atrial fibrillation is associated with sudden cardiac death: a systematic review and meta-analysis. *Journal of interventional cardiac electrophysiology : an international journal of arrhythmias and pacing* 2018; 51: 91-104. 2018/01/15. DOI: 10.1007/s10840-017-0308-9.

135. Briasoulis A, Mallikethi-Reddy S, Palla M, et al. Myocardial fibrosis on cardiac magnetic resonance and cardiac outcomes in hypertrophic cardiomyopathy: a meta-analysis. *Heart* 2015; 101: 1406-1411. 2015/06/11. DOI: 10.1136/heartjnl-2015-307682.

136. Norrish G, Cantarutti N, Pissaridou E, et al. Risk factors for sudden cardiac death in childhood hypertrophic cardiomyopathy: A systematic review and meta-analysis. *Eur J Prev Cardiol* 2017; 24: 1220-1230. 2017/05/10. DOI: 10.1177/2047487317702519.

137. Kuruvilla S, Adenaw N, Katwal AB, et al. Late gadolinium enhancement on cardiac magnetic resonance predicts adverse cardiovascular outcomes in nonischemic cardiomyopathy: a systematic review and meta-analysis. *Circ Cardiovasc Imaging* 2014; 7: 250-258. 2013/12/24. DOI: 10.1161/circimaging.113.001144.

138. Peck KY, Lim YZ, Hopper I, et al. Medical therapy versus implantable cardioverter -defibrillator in preventing sudden cardiac death in patients with left ventricular systolic dysfunction and heart failure: a meta-analysis of > 35,000 patients. *Int J Cardiol* 2014; 173: 197-203. 2014/03/19. DOI: 10.1016/j.ijcard.2014.02.014.

139. Cheng YJ, Nie XY, Chen XM, et al. The Role of Macrolide Antibiotics in Increasing Cardiovascular Risk. *J Am Coll Cardiol* 2015; 66: 2173-2184. 2015/11/14. DOI: 10.1016/j.jacc.2015.09.029.

140. Cheng YJ, Lin XX, Ji CC, et al. Role of Early Repolarization Pattern in Increasing Risk of Death. *J Am Heart Assoc* 2016; 5 2016/09/28. DOI: 10.1161/jaha.116.003375.

141. Cheng YJ, Li ZY, Yao FJ, et al. Early repolarization is associated with a significantly increased risk of ventricular arrhythmias and sudden cardiac death in patients with structural heart diseases. *Heart Rhythm* 2017; 14: 1157-1164. 2017/04/19. DOI: 10.1016/j.hrthm.2017.04.022.

142. Konety SH, Koene RJ, Norby FL, et al. Echocardiographic Predictors of Sudden Cardiac Death: The Atherosclerosis Risk in Communities Study and Cardiovascular Health Study. *Circ Cardiovasc Imaging* 2016; 9 2016/08/09. DOI: 10.1161/circimaging.115.004431.

143. Le HH, El-Khatib C, Mombled M, et al. Impact of Aldosterone Antagonists on Sudden Cardiac Death Prevention in Heart Failure and Post-Myocardial Infarction Patients: A Systematic Review and Meta-Analysis of Randomized Controlled Trials. *PLoS One* 2016; 11: e0145958. 2016/02/20. DOI: 10.1371/journal.pone.0145958.

144. Renoux C, Dell'Aniello S, Khairy P, et al. Ventricular tachyarrhythmia and sudden cardiac death with domperidone use in Parkinson's disease. *British Journal of Clinical Pharmacology* 2016; 82: 461-472. DOI: <https://doi.org/10.1111/bcp.12964>.

145. Pelliccia F, Pasceri V, Limongelli G, et al. Long-term outcome of nonobstructive versus obstructive hypertrophic cardiomyopathy: A systematic review and meta-analysis. *Int J Cardiol* 2017; 243: 379-384. 2017/07/28. DOI: 10.1016/j.ijcard.2017.06.071.

146. Aune D, Schlesinger S, Norat T, et al. Diabetes mellitus and the risk of sudden cardiac death: A systematic review and meta-analysis of prospective studies. *Nutrition, metabolism, and cardiovascular diseases : NMCD* 2018; 28: 543-556. 2018/05/08. DOI: 10.1016/j.numecd.2018.02.011.

147. Duan X, Li J, Zhang Q, et al. Prognostic value of late gadolinium enhancement in dilated cardiomyopathy patients: a meta-analysis. *Clin Radiol* 2015; 70: 999-1008. 2015/06/28. DOI: 10.1016/j.crad.2015.05.007.

148. Salvo F, Pariente A, Shakir S, et al. Sudden cardiac and sudden unexpected death related to antipsychotics: A meta-analysis of observational studies. *Clin Pharmacol Ther* 2016; 99: 306-314. 2015/08/15. DOI: 10.1002/cpt.250.

149. Taverny G, Mimouni Y, LeDigarcher A, et al. Antihypertensive pharmacotherapy for prevention of sudden cardiac death in hypertensive individuals. *Cochrane Database Syst Rev* 2016; 3: Cd011745. 2016/03/11. DOI: 10.1002/14651858.CD011745.pub2.

150. Kolodziejczak M, Andreotti F, Kowalewski M, et al. Implantable Cardioverter-Defibrillators for Primary Prevention in Patients With Ischemic or Nonischemic Cardiomyopathy: A Systematic Review and Meta-analysis. *Annals of internal medicine* 2017; 167: 103-111. 2017/06/21. DOI: 10.7326/m17-0120.

151. Siddiqui WJ, Aggarwal S, Rafique M, et al. Prophylactic use of the implantable cardioverter-defibrillator and its effect on the long-term survival, cardiovascular and sudden cardiac death in nonischemic cardiomyopathy patients-a systematic review and meta-analysis. *Heart Fail Rev* 2018; 23: 181-190. 2018/01/31. DOI: 10.1007/s10741-018-9671-6.

152. Gama F, Ferreira J, Carmo J, et al. Implantable Cardioverter-Defibrillators in Trials of Drug Therapy for Heart Failure: A Systematic Review and Meta-Analysis. *J Am Heart Assoc* 2020; 9: e015177. 2020/04/16. DOI: 10.1161/jaha.119.015177.

153. Al-Gobari M, Al-Aqeel S, Gueyffier F, et al. Effectiveness of drug interventions to prevent sudden cardiac death in patients with heart failure and reduced ejection fraction: an overview of systematic reviews. *BMJ Open* 2018; 8: e021108. DOI: 10.1136/bmjopen-2017-021108.

154. Aune D, Schlesinger S, Norat T, et al. Tobacco smoking and the risk of sudden cardiac death: a systematic review and meta-analysis of prospective studies. *Eur J Epidemiol* 2018; 33: 509-521. 2018/02/09. DOI: 10.1007/s10654-017-0351-y.

155. Aune D, Schlesinger S, Norat T, et al. Body mass index, abdominal fatness, and the risk of sudden cardiac death: a systematic review and dose-response meta-analysis of prospective studies. *Eur J Epidemiol* 2018; 33: 711-722. 2018/02/09. DOI: 10.1007/s10654-017-0353-9.

156. Ganesan AN, Gunton J, Nucifora G, et al. Impact of Late Gadolinium Enhancement on mortality, sudden death and major adverse cardiovascular events in ischemic and nonischemic cardiomyopathy: A systematic review and meta-analysis. *Int J Cardiol* 2018; 254: 230-237. 2018/02/07. DOI: 10.1016/j.ijcard.2017.10.094.

157. Yue P, Jing S, Liu L, et al. Association between mitochondrial DNA copy number and cardiovascular disease: Current evidence based on a systematic review and meta-analysis. *PLoS One* 2018; 13: e0206003. 2018/11/08. DOI: 10.1371/journal.pone.0206003.

158. Venkatesh P, Evans AT, Maw AM, et al. Predictors of Late Mortality in D&#x2010;Transposition of the Great Arteries After Atrial Switch Repair: Systematic Review and Meta&#x2010;Analysis. *Journal of the American Heart Association* 2019; 8: e012932. DOI: doi:10.1161/JAHA.119.012932.

159. Fernandes GC, Fernandes ADF, Rivera M, et al. A meta-analysis of arrhythmia endpoints in randomized controlled trials of transendocardial stem cell injections for chronic ischemic heart disease. *Journal of Cardiovascular Electrophysiology* 2019; 30: 2492-2500. DOI: <https://doi.org/10.1111/jce.14185>.

160. Liang Z, Zhu J, Chen L, et al. Is androgen deprivation therapy for prostate cancer associated with cardiovascular disease? A meta-analysis and systematic review. *Andrology* 2020; 8: 559-574. DOI: <https://doi.org/10.1111/andr.12731>.

161. Chen H, Deng Y and Li S. Relation of Body Mass Index Categories with Risk of Sudden Cardiac Death. *Int Heart J* 2019; 60: 624-630. 2019/05/21. DOI: 10.1536/ihj.18-155.

162. Pan H, Hibino M, Kobeissi E, et al. Blood pressure, hypertension and the risk of sudden cardiac death: a systematic review and meta-analysis of cohort studies. *Eur J Epidemiol* 2020; 35: 443-454. 2019/12/26. DOI: 10.1007/s10654-019-00593-4.

163. Yang F, Wang J, Li W, et al. The prognostic value of late gadolinium enhancement in myocarditis and clinically suspected myocarditis: systematic review and meta-analysis. *Eur Radiol* 2020; 30: 2616-2626. 2020/02/11. DOI: 10.1007/s00330-019-06643-5.

164. Aune D, Schlesinger S, Hamer M, et al. Physical activity and the risk of sudden cardiac death: a systematic review and meta-analysis of prospective studies. *BMC cardiovascular disorders* 2020; 20: 318. 2020/07/08. DOI: 10.1186/s12872-020-01531-z.

165. Kamp NJ, Chery G, Kosinski AS, et al. Risk stratification using late gadolinium enhancement on cardiac magnetic resonance imaging in patients with hypertrophic cardiomyopathy: A systematic review and meta-analysis. *Prog Cardiovasc Dis* 2020 2020/11/11. DOI: 10.1016/j.pcad.2020.11.001.

166. Bytyçi I, Nistri S, Mörner S, et al. Alcohol Septal Ablation versus Septal Myectomy Treatment of Obstructive Hypertrophic Cardiomyopathy: A Systematic Review and Meta-Analysis. *J Clin Med* 2020; 9 2020/09/27. DOI: 10.3390/jcm9103062.

167. Fernandes GC, Fernandes A, Cardoso R, et al. Association of SGLT2 inhibitors with arrhythmias and sudden cardiac death in patients with type 2 diabetes or heart failure: A meta-analysis of 34 randomized controlled trials. *Heart Rhythm* 2021. DOI: <https://doi.org/10.1016/j.hrthm.2021.03.028>.
